# Genetic variation in the MacAB-TolC efflux pump influences pathogenesis of invasive *Salmonella* isolates from Africa

**DOI:** 10.1371/journal.ppat.1008763

**Published:** 2020-08-24

**Authors:** Jared D. Honeycutt, Nicolas Wenner, Yan Li, Susan M. Brewer, Liliana M. Massis, Sky W. Brubaker, Phoom Chairatana, Siân V. Owen, Rocío Canals, Jay C. D. Hinton, Denise M. Monack

**Affiliations:** 1 Department of Microbiology and Immunology, Stanford University School of Medicine, Stanford, California, United States of America; 2 Institute of Integrative Biology, University of Liverpool, Liverpool, United Kingdom; 3 Department of Microbiology, Faculty of Medicine Siriraj Hospital, Mahidol University, Bangkok, Thailand; 4 Department of Biomedical Informatics, Harvard Medical School, Boston, Massachusetts, United States of America; University of California Davis School of Medicine, UNITED STATES

## Abstract

The various sub-species of *Salmonella enterica* cause a range of disease in human hosts. The human-adapted *Salmonella* enterica serovar Typhi enters the gastrointestinal tract and invades systemic sites to cause enteric (typhoid) fever. In contrast, most non-typhoidal serovars of *Salmonella* are primarily restricted to gut tissues. Across Africa, invasive non-typhoidal *Salmonella* (iNTS) have emerged with an ability to spread beyond the gastrointestinal tract and cause systemic bloodstream infections with increased morbidity and mortality. To investigate this evolution in pathogenesis, we compared the genomes of African iNTS isolates with other *Salmonella enterica* serovar Typhimurium and identified several *macA* and *macB* gene variants unique to African iNTS. MacAB forms a tripartite efflux pump with TolC and is implicated in *Salmonella* pathogenesis. We show that *macAB* transcription is upregulated during macrophage infection and after antimicrobial peptide exposure, with *macAB* transcription being supported by the PhoP/Q two-component system. Constitutive expression of *macAB* improves survival of *Salmonella* in the presence of the antimicrobial peptide C18G. Furthermore, these *macAB* variants affect replication in macrophages and influence fitness during colonization of the murine gastrointestinal tract. Importantly, the infection outcome resulting from these *macAB* variants depends upon both the *Salmonella* Typhimurium genetic background and the host gene *Nramp1*, an important determinant of innate resistance to intracellular bacterial infection. The variations we have identified in the MacAB-TolC efflux pump in African iNTS may reflect evolution within human host populations that are compromised in their ability to clear intracellular *Salmonella* infections.

## Author summary

*Salmonella* Typhimurium will generally cause acute gut infections in humans. However, *S*. Typhimurium strains causing severe, systemic infections have emerged in sub-Saharan Africa and are phylogenetically distinct from other *S*. Typhimurium strains. Our comparative genomic analysis revealed *S*. Typhimurium sequence-type 313 (ST313) from Africa have notable sequence variations within the *macA* and *macB* genes. These genes are already known to play a role in *Salmonella* pathogenesis and are otherwise conserved in *Salmonella* and many other Gram-negative bacteria. We show that regulation of *macAB* transcription depends, in part, on the key *Salmonella* virulence system PhoP/Q and that expression of MacAB improves *Salmonella* resistance to an antimicrobial peptide. African *macAB* variants interfere with this antimicrobial peptide resistance function and can alter *Salmonella* replication within macrophages. Using competitive infection experiments in mice, we see that these *macAB* variants influence fitness in the mammalian gut and systemic sites, with African *S*. Typhimurium reliant upon its *macAB* genotype for systemic infection of susceptible hosts. These results suggest that the evolution of African *S*. Typhimurium has been shaped by human populations with impaired ability to control intracellular *Salmonella* infections.

## Introduction

*Salmonella* infections continue to be a significant challenge for human health. With an estimated 95 million annual cases, non-typhoidal *Salmonella* (NTS) infection is typically characterized by severe but self-resolving gastroenteritis in otherwise healthy people [1–3]. Typhoid and paratyphoid fever cases number more than 14 million annually and are characterized by invasive, bloodstream infection by *Salmonella* serovars Typhi and Paratyphi, respectively [4]. Risk for typhoid disease remains high in geographic areas with inadequate sanitation infrastructure as the infectious cycle relies on human-to-human transmission. While some humans can become asymptomatic chronic carriers, untreated typhoid fever is often fatal [3]. With appropriate treatment, the 1% case mortality of typhoid fever is similar to that of gastroenteritis associated with NTS [2,4,5].

Invasive non-typhoidal *Salmonella* (iNTS) isolates belonging to the serovars Typhimurium and Enteritidis have caused major disease outbreaks within sub-Saharan Africa [6–9]. These African iNTS isolates are associated with systemic infections, and particularly high case mortality in children less than 5 years of age, the elderly, and those with comorbidities such as HIV and malaria [6,10]. Although typhoid infections outnumber iNTS infections by over 25-fold globally, iNTS caused nearly half as many deaths with overall mortality rates above 14% [5]. In Africa, iNTS was responsible for 49,600 deaths in 2017 [5]. There is great need to further understand iNTS pathogenesis and epidemiology in order to improve diagnosis and clinical outcomes for these increasingly antibiotic resistant infections [11–13].

By multi-locus sequence typing, many *Salmonella enterica* serovar Typhimurium (*S*. Typhimurium) gastrointestinal isolates are classified as sequence-type 19 (“ST19”), while African *S*. Typhimurium associated with invasive disease belong mainly to sequence-type 313 (“ST313”) [7,9]. ST313 isolates have also been observed in the UK and Brazil, though African ST313 form a distinct lineage [14–16]. Comparative genomic analysis has identified numerous changes in African ST313 lineages. While they have acquired unique prophages, plasmids, and antibiotic resistance genes, African ST313 also display gene degradation events that impair the ability of these isolates to survive outside of mammalian hosts [13,17–23]. Further gene degradation events have been shown to alter invasive and immune stimulating behavior in experimental animal models, supporting the view that African iNTS strains are evolving from causing strictly enteropathogenic disease to causing invasive disease in human hosts [15,24–26].

In our comparative analysis of *S*. Typhimurium ST313 lineage isolates with other *Salmonella* genomes we observed specific changes at the *macAB* locus in ST313 lineages. In Gram-negative bacteria, MacAB forms a tripartite channel with the outer membrane protein TolC to efflux various antimicrobial compounds as well as endogenous molecules and toxins [27–31]. As an ABC-type efflux pump, hydrolysis of cytoplasmic ATP by MacB directly drives movement of MacA and TolC to translocate molecules from the periplasm into the extracellular space [32,33]. The naming of *macA* and *macB* (previously annotated as *ybjY* and *ybjZ*) references their ability to confer resistance to macrolide antibiotics when overexpressed together from a plasmid [34], though in standard laboratory culture conditions *macAB* is not expressed in *Salmonella* [35,36]. In clinical isolates of other bacteria, increased expression of *macAB* homologues increases resistance to antimicrobial peptides such as polymyxins [37]. The fact that MacAB has a virulence role in animal models of oral *Salmonella* infection [35,38] prompted us to explore how African ST313-associated *macAB* gene variants might influence pathogenesis.

## Results

### Lineage-specific variation of the *macAB* locus in invasive African *S*. Typhimurium ST313 isolates

**Fig 1:**
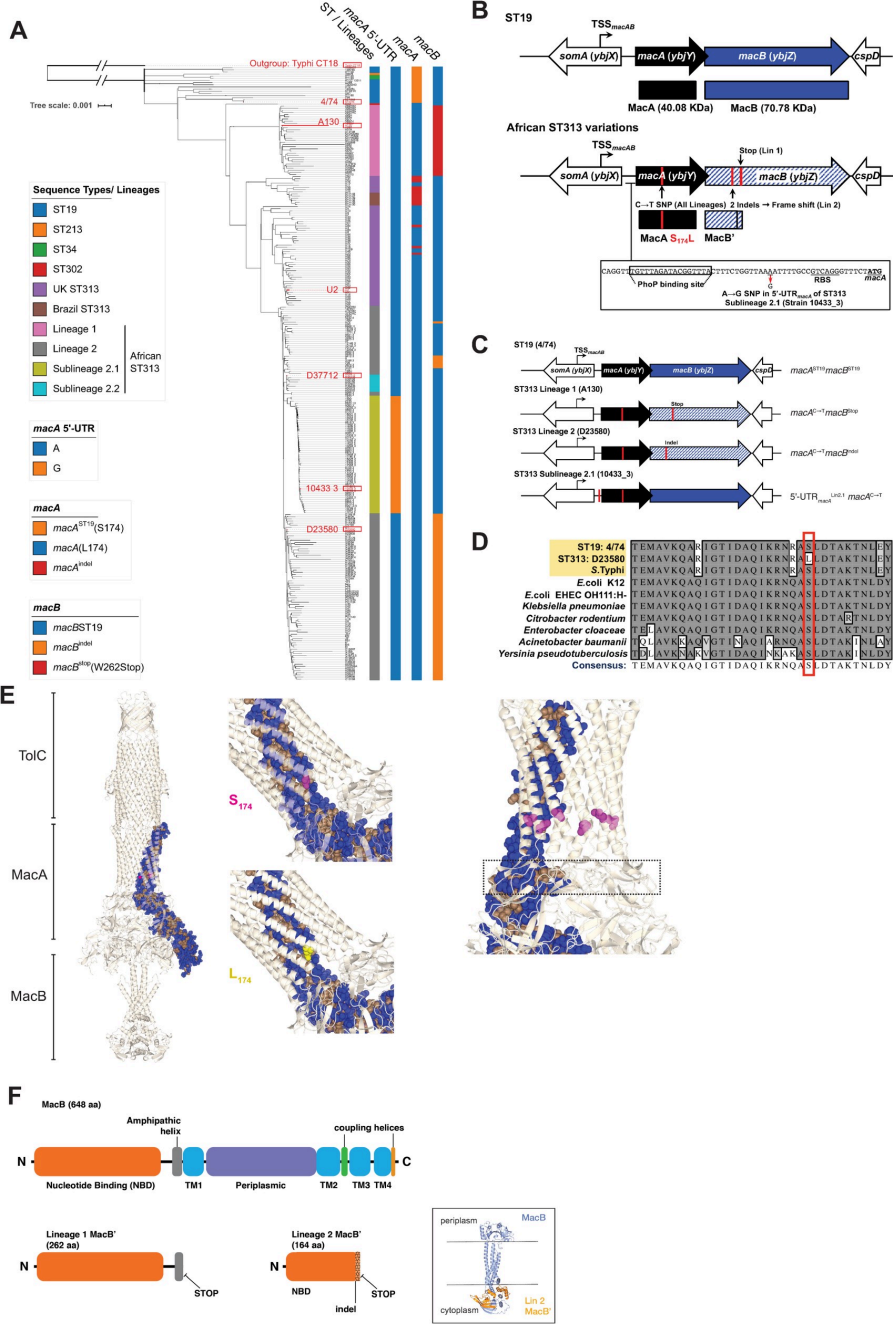
Variation at the *macAB* locus in African *S*. Typhimurium ST313. (A) Phylogeny emphasizing all available ST313 isolates from Africa, the UK, and Brazil, with sequence type and lineage membership indicated by first vertical colored bar. *Salmonella* Typhi CT18 was used as an outgroup for tree construction. Presence of *macAB* variants are shown by corresponding colored bars. Representative isolates of each lineage are highlighted in red. (B) The *macAB* genomic locus with SNP locations and their effects on MacA and MacB proteins. The proximal upstream region of the *macA* start codon indicating the PhoP-box characterized by Nishino *et al* [35] and the 5’-UTR_*macA*_^Lin2.1^ SNP identified by Van Puyvelde *et al* [13] (B, bottom). 5’-UTR = 5’ untranslated region. TSS_*macAB*_ = *macAB* transcription start site, 504 bases upstream of *macA* start codon. RBS = ribosome binding site. (C) *macAB* genomic locus highlighting variant combinations that pertain to each lineage. (D) Alignment showing conservation of the amino acid sequence surrounding the S_174_ residue of MacA with the ST313 S_174_L mutation boxed in red. (E) Overlay of 4/74 MacA predicted structure onto *E*. *coli* MacAB-TolC (PDB 5NIK), with residues colored blue and tan for hydrophilic and hydrophobic side chains, respectively. S_174_ highlighted in magenta (E, left and middle top) and L_174_ in yellow (E, middle bottom). S_174_ highlighted in all chains of the MacA hexamer, with the putative channel gating ring [33] in boxed outline (E, right). (F) *E*. *coli* MacB domain architecture from [32] (E, top) with *macB*^W262Stop^ and *macB*^indel^ truncations of MacB (E, bottom). Overlay of truncated MacB structure (orange) onto *E*. *coli* MacB (blue) (F, inset). 4/74 MacA prediction by Phyre2 [39]. Structural diagrams generated with CCP4 [40] using the operation superpose [41].

Comparative analysis of all currently available genomes of ST313 isolates of *Salmonella enterica* serovar Typhimurium identified several genomic changes at the *macAB* locus that differed from other gastroenteritis-associated *S*. Typhimurium (Fig 1A). Although some UK and Brazilian ST313 isolates carry an indel in the *macA* gene, this variant is not present in African isolates from ST313 lineages (1, 2) or sublineages (2.1, 2.2) that are associated with invasive disease (Fig 1A). Instead, all other ST313 lineage isolates in our analysis carry a C➝T non-synonymous SNP within *macA* (Fig 1A), which replaces Serine (S_174_) with Leucine (L_174_) (Fig 1B and 1C). Alignment of this sub-region of MacA shows that the Serine residue is highly conserved amongst other Gram-negative bacterial genera (Fig 1D). The structure of the *Escherichia coli* (*E*. *coli*) MacAB-TolC complex has been solved [33]; assuming an analogous overall structure in *S*. Typhimurium, the hydrophilic Serine_174_ residue faces the channel interior, residing beyond the proposed gating ring and in series with other hydrophilic residues that form the interior surface of the MacA channel [33] (Fig 1E). We hypothesized that the ST313-associated mutation of this conserved Serine residue to the hydrophobic amino acid Leucine altered the function of the MacAB-TolC channel, particularly since this mutation would be repeated around the interior of the fully-assembled hexameric MacA channel (Fig 1E, right).

*S*. Typhimurium ST313 lineage 1 isolates contain a nonsense mutation in *macB* [19] changing the codon for W262 of MacB to a stop codon; this change lies within the amphipathic helix that precedes the first transmembrane domain [32], leading to the production of a truncated MacB (Fig 1F) [32]. Furthermore, a two-nucleotide insertion (an indel) that results in a frameshift in the *macB* gene was found in about half of the *S*. Typhimurium ST313 lineage 2 isolates, including the reference strain D23580. This pseudogenization event created a stop codon that truncates the MacB protein (Fig 1F). This truncation interrupts the N-terminal ATP-binding domain and prevents translation of the transmembrane domains that extend from the cytoplasm into the periplasmic space (Fig 1F); assuming an analogous structure to *E*. *coli* MacB, the truncated MacB protein in ST313 Lineage 2 is predicted to be unable to interact with MacA in the context of the MacAB-TolC efflux pump [32].

The recently described African *S*. Typhimurium ST313 sublineage 2.1 [13] retains the *macA*^C➝T^ SNP associated with other ST313 while also having an A➝G SNP in the 5’ untranslated region (5’-UTR) of *macA* (Fig 1B). Sublineage 2.1 and 2.2 isolates do not carry the *macB* mutations associated with lineage 1 or 2 isolates (Fig 1A).

The presence of multiple variations at the *macAB* locus of African ST313 suggested reductive or adaptive evolution has occurred [25]. We focused on characterizing *macAB* variants found in the most recently isolated African *S*. Typhimurium ST313 lineages 2 and sublineage 2.1 that are associated with invasive disease (Fig 1C). For clarity, these mutations are designated: *macA*^C➝T^ for the non-synonymous SNP shared across all ST313 lineages; *macB*^indel^ for the SNP present in ST313 lineage 2 that introduces multiple stop codons due to a frameshift and truncates the MacB protein; and 5’-UTR_*macA*_^Lin2.1^ for the A➝G SNP within the 5’-UTR of *macA* of ST313 sublineage 2.1 isolates. We use *macA*^ST19^ and *macB*^ST19^ to refer to the alleles that are carried by *S*. Typhimurium ST19.

### Expression of *macAB* is promoted by PhoP in *Salmonella* Typhimurium

The *macAB* (*ybjY-ybjZ*) genes are operonic with the transcriptional start site (TSS) located 504 nucleotides upstream of the translational start of the *macA* gene [36]. This is a particularly long 5’-UTR, and regions of this type have previously been shown to play important regulatory roles in *Salmonella* [42,43].

Since previous studies suggested that *macAB* is important for *S*. Typhimurium virulence [35,38], we wanted to clarify how expression of this locus is regulated. In *Salmonella* the two-component system PhoP/Q senses low magnesium, acidic pH, and antimicrobial peptide disturbance of the inner membrane. PhoP up-regulates a set of genes that increase cellular resistance to antimicrobial peptides and promote survival in macrophages [44–48]. Previously, PhoP was shown to physically bind to a PhoP-box upstream of the *macA* coding sequence, with the authors concluding that PhoP represses *macAB* transcription [35].

**Fig 2:**
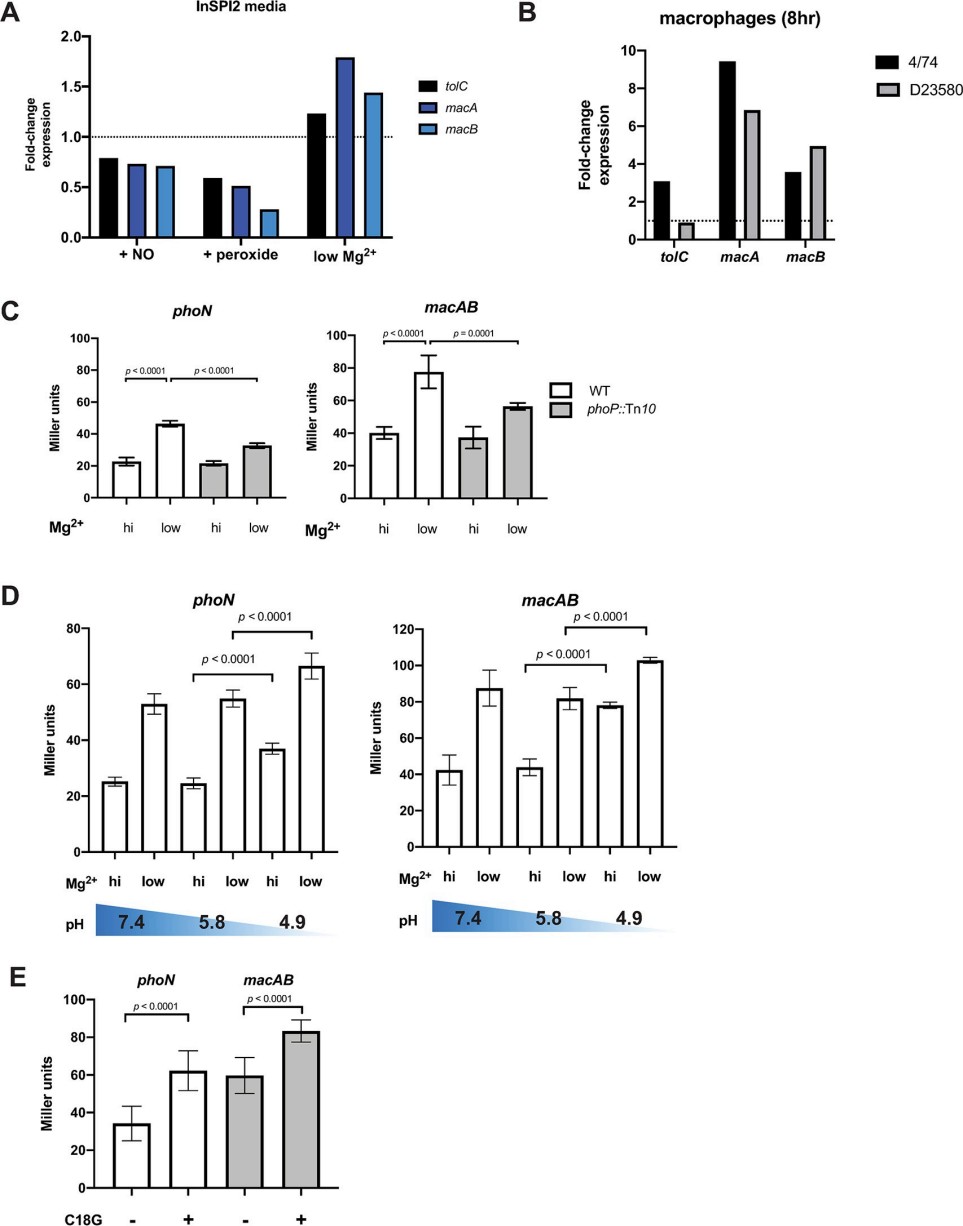
*macAB* is a PhoP-regulated gene in *Salmonella* Typhimurium. (A) Gene expression in InSPI2 media in response to environmental stimuli, fold-change relative to InSPI2 medium alone. (B) *S*. Typhimurium intramacrophage gene expression measured from RAW264.7 macrophages after 8 hours of infection, fold-change relative to expression in LB at early stationary phase. Panels A and B show RNA-seq data extracted from the SalComD23580, SalComMac, and SalComRegulon databases reported previously [18,36,49]. (C) ST19 4/74 and 4/74 *phoP* null mutants (*phoP*::Tn*10*) with chromosomally integrated *lacZY* transcriptional fusions for either *phoN* and *macAB* were grown to mid-exponential phase in N minimal medium, pH 7.4 and high (10mM) MgCl_2_ before transfer to the same or low (10μM) MgCl_2_ media and growth for 90 minutes. β-galactosidase production was measured by a kinetic Miller assay. (D) At mid-exponential phase, transcriptional fusion strains were shifted from pH 7.4 and 10mM MgCl_2_ to media buffered to the indicated pH with high (10mM) or low (10μM) MgCl_2_ and grown for 90 minutes. (E) Transcriptional fusion strains at mid-exponential phase were exposed to the antimicrobial peptide C18G (5μg/mL) in N minimal medium at pH 7.4 with 1mM MgCl_2_ and grown for 90 minutes. Data are from three repeat experiments. ANOVA with Tukey post-test (C, D), or t-test (E); bar = mean; error = standard deviation.

We previously published the RNA-seq-based transcriptomic profiles of *S*. Typhimurium ST19 strain 4/74 during growth in multiple *in vitro* conditions and during intramacrophage replication [36,49]. The major pathogenicity locus SPI-2 is important for survival of *Salmonella* in mammalian phagocytic cells, and expression of SPI-2 genes can be induced by defined media that mimic some conditions of the vacuolar environment (InSPI2). Our published data show that *S*. Typhimurium 4/74 increases transcription of *macA* and *macB* in low magnesium InSPI2 medium, consistent with a role for stimuli sensed by PhoP/Q in promoting *macAB* expression (Fig 2A). More recently, we published the transcriptomic profiles of the *macAB* genes of *S*. Typhimurium ST313 strain D23580 using the same environmental conditions, including intra-macrophage replication [18], summarized at https://tinyurl.com/macAB-SalCom474-D23. In data sets from both ST19 isolate 4/74 and ST313 lineage 2 isolate D23580, the *macAB* transcript is upregulated during replication in RAW264.7 macrophage-like cells, compared to early stationary phase (ESP) growth in LB medium (Fig 2B). Furthermore, we previously observed that deletion of *phoPQ* reduced *macAB* transcription by about half in InSPI2 medium [50].

To examine *macAB* transcriptional regulation more closely, we created chromosomal *lacZY* transcriptional fusions in *S*. Typhimurium ST19 isolate 4/74 driven by the endogenous *phoN* or *macAB* promoters. We grew these reporter strains overnight in defined N minimal medium at pH 7.4 with high (10mM) magnesium, a condition that represses PhoP activity [51]. After subculture and growth to mid-exponential phase, we shifted cells to low (10μM) magnesium medium, a treatment that up-regulates expression of the PhoP-regulated gene *phoN* [52]. We found that β-galactosidase levels increased for both *phoN*::*lacZY* (positive control) as well as *macAB*::*lacZY* when cells were shifted to low magnesium medium (Fig 2C). Furthermore, the up-regulation of these genes was impaired when *phoP* null mutants were shifted to low magnesium medium (Fig 2C). In addition, when cells with the *phoN* and *macAB* transcriptional fusions were shifted to increasingly acidic conditions (from pH 5.8 to pH 4.9), levels of β-galactosidase activity were significantly increased (Fig 2D), in agreement with previous reports that acidic pH stimulates expression of PhoP/Q-dependent genes [53,54].

Cationic antimicrobial peptides induce expression of PhoP-regulated genes [52] and play bacteriostatic and bactericidal roles during *Salmonella* infection of macrophages [55]. To test whether *macAB* gene expression is upregulated by antimicrobial peptides, we grew the *phoN* and *macAB* transcriptional fusion strains in moderate (1mM) magnesium followed by treatment with a sub-inhibitory concentration of the salt-insensitive, cationic antimicrobial peptide C18G [52]. Exposure to C18G induced higher β-galactosidase activity in the *phoN* and *macAB* transcriptional fusion strains (Fig 2E). These experiments collectively show that *macAB* transcription is facilitated by PhoP under biologically relevant conditions.

### African *S*. Typhimurium ST313 *macAB* variants influence replication in macrophages

We next wanted to determine if the genetic changes in the *macAB* locus of the ST313 lineages commonly associated with invasive disease in Africa had functional consequences during infection. We focused our experiments on lineage 2 and sublineage 2.1 *macAB* variants, reasoning that the lineage 1 *macB*^STOP^ would have effects that are similar to the lineage 2 *macB*^indel^. Furthermore, ST313 lineage 1 isolates are no longer causing a clinical problem in Africa [12]. To ensure otherwise native regulation of the *macAB* genes, we introduced marker-less nucleotide changes directly into the *macAB* locus of the ST19 isolate 4/74 or the ST313 lineage 2 isolate D23580. The 4/74 *macA*^C➝T^ mutant was made to test the impact of this SNP alone, the 4/74 *macA*^C➝T^ *macB*^indel^ mutant to represent the lineage 2 *macAB* genotype, and the 4/74 5’-UTR_*macA*_^Lin2.1^ *macA*^C➝T^ mutant to represent the lineage 2.1 *macAB* genotype (Fig 1C). We additionally made a 4/74 5’-UTR_*macA*_^Lin2.1^ mutant to test whether this SNP alone affects pathogenesis.

Similarly, we modified D23580 to test the role of individual *macAB* SNPs in modulating virulence. The ST313 lineage 2 *macB*^indel^ was first removed, yielding D23580 *macA*^C➝T^ *macB*^ST19^. This was followed by alteration of the *macA*^C➝T^ SNP, creating a D23580 strain with the full ST19 genotype (D23580 *macA*^ST19^*macB*^ST19^). We confirmed that our engineered strains carried the desired nucleotide modifications, with no unintended mutations elsewhere in the chromosome, by whole genome sequencing (see S2 Table and Materials and Methods).

**Fig 3:**
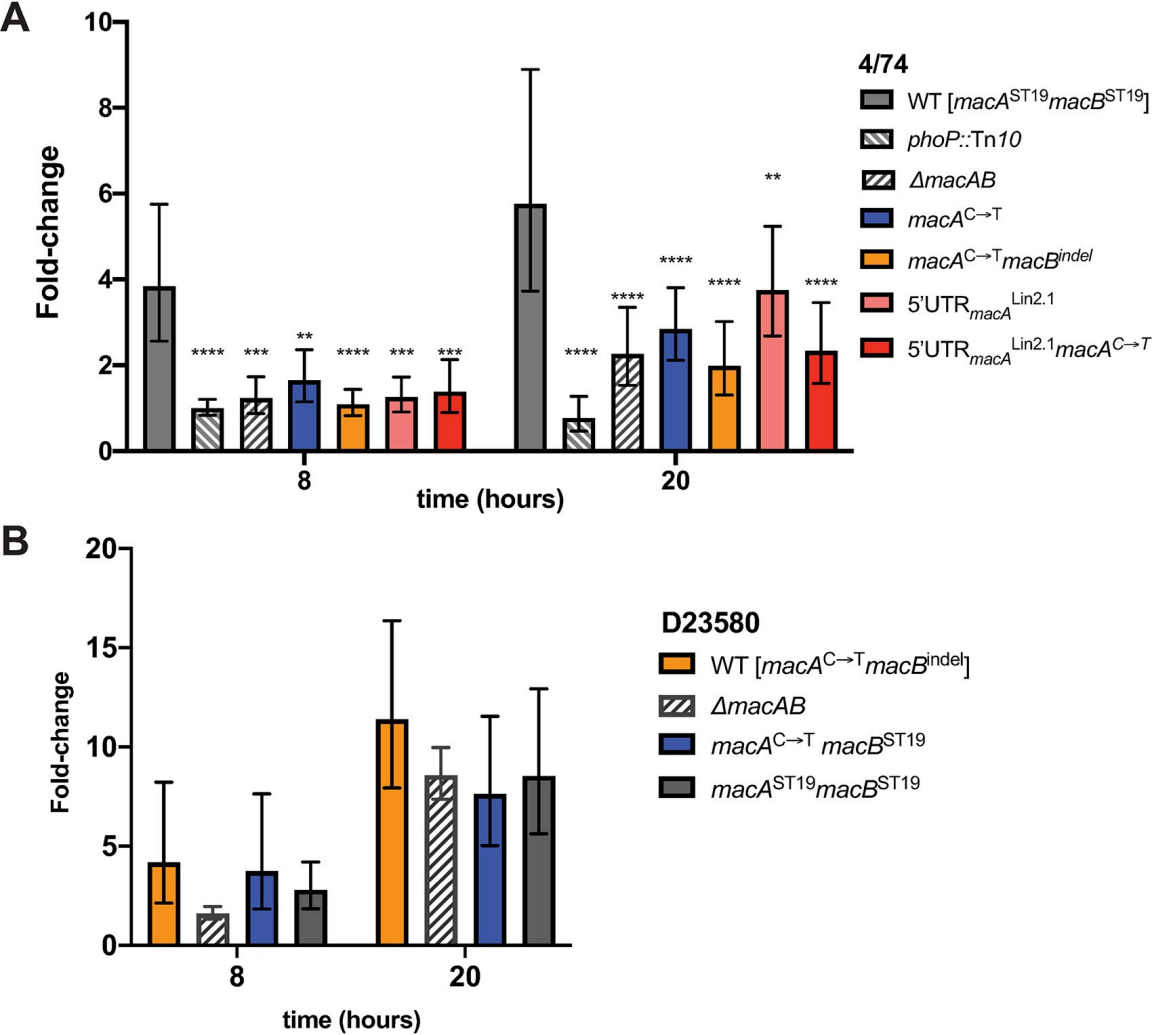
*S*. Typhimurium ST313 *macAB* variants impede ST19 4/74 replication in RAW macrophages. RAW264.7 cells were infected at an MOI of 10 with bacteria from overnight stationary phase cultures. Replication was assessed by plating bacteria at the indicated timepoints with fold-replication calculated relative to CFU/well at *t* = 0 as described in Materials and Methods. (A) *S*. Typhimurium ST19 4/74 with derived mutants, and (B) ST313 lineage 2 isolate D23580 with derived mutants. Two-way ANOVA (time, strain) with Dunnett’s post-test comparing each mutant to the parent strain; bar = geometric mean; error bars = geometric standard deviation. ** = *p* < 0.01, *** = *p* < 0.001, **** = *p <* 0.0001.

Previous studies have shown that *S*. Typhimurium ST19 strains 14028S and SL1344 with deletions of *macAB* replicate poorly within mouse macrophages [38,56]. We infected the murine RAW264.7 macrophage cell line with a range of our marker-less *macAB* mutants to assess *Salmonella* intracellular replication. As expected, we found that ST19 4/74 *phoP* and *macAB* null mutants showed significantly reduced replication in macrophages compared to parental 4/74 (Fig 3A). Replication of the 4/74 *macA*^C➝T^ mutant was significantly lower than the parental 4/74 and similar to replication of the *macAB* null mutant (Fig 3A) showing this ST313-associated SNP likely impairs MacAB-TolC functionality during intramacrophage replication. The 4/74 *macA*^C➝T^ *macB*^indel^ mutant (ST313 lineage 2 genotype) also showed lower replication than parental 4/74 (Fig 3A). Interestingly, the 5’-UTR_*macA*_^Lin2.1^ SNP alone significantly reduced replication of 4/74, while the 4/74 5’-UTR_*macA*_^Lin2.1^ *macA*^C➝T^ mutant with the full ST313 sublineage 2.1 genotype replicated at a level similar to the *macAB* null mutant (Fig 3A).

We also assessed replication of *S*. Typhimurium D23580, the reference ST313 lineage 2 African isolate, in murine RAW264.7 macrophages. D23580 replicated extensively in RAW cells to a higher level than 4/74 (Fig 3A and 3B), as previously reported [17]. We made marker-less point mutations in D23580 to change the *macA*^C➝T^ and *macB*^indel^ SNPs to the respective ST19 alleles. The D23580 *macA*^C➝T^*macB*^ST19^ and D23580 *macA*^ST19^*macB*^ST19^ mutants showed no change in fold-replication in this experimental setting when compared to the parent D23580 (Fig 3B).

Taken together, these results show the *macAB*-associated SNPs of *S*. Typhimurium ST313 lineages do have phenotypic consequences in macrophage replication, though the replication effect may be epistatic. The ST19 strain 4/74 shows reduced levels of intra-macrophage replication when *macAB* is modified to the various ST313 genotypes, suggesting that these SNP mutations impair MacAB-TolC function. In contrast, an African ST313 lineage 2 isolate maintains its level of intra-macrophage replication irrespective of *macAB* genotype. This implies that D23580 has a *macAB*-independent mechanism for its enhanced replication phenotype in RAW macrophage-like cells.

### ST19 *macAB* provides resistance to the cationic antimicrobial peptide C18G

Previous studies have shown that MacAB homologues can contribute to increased resistance to antimicrobial peptides in other bacteria [32]. In *E*. *coli*, the TolC-dependent secretion of the helical, amphiphilic peptide enterotoxin II is facilitated by MacAB, but not by other TolC-interacting partners [57]. Additionally, another ABC-type efflux pump (MtrCDE) improves *Neisseria* resistance to both macrolide antibiotics and antimicrobial peptides [58]. Since we showed *macAB* expression in *S*. Typhimurium is regulated by the PhoP/Q system that is important for antimicrobial peptide resistance, we hypothesized that MacAB of *Salmonella* would also support replication in the presence of amphiphilic cationic antimicrobial peptides.

**Fig 4:**
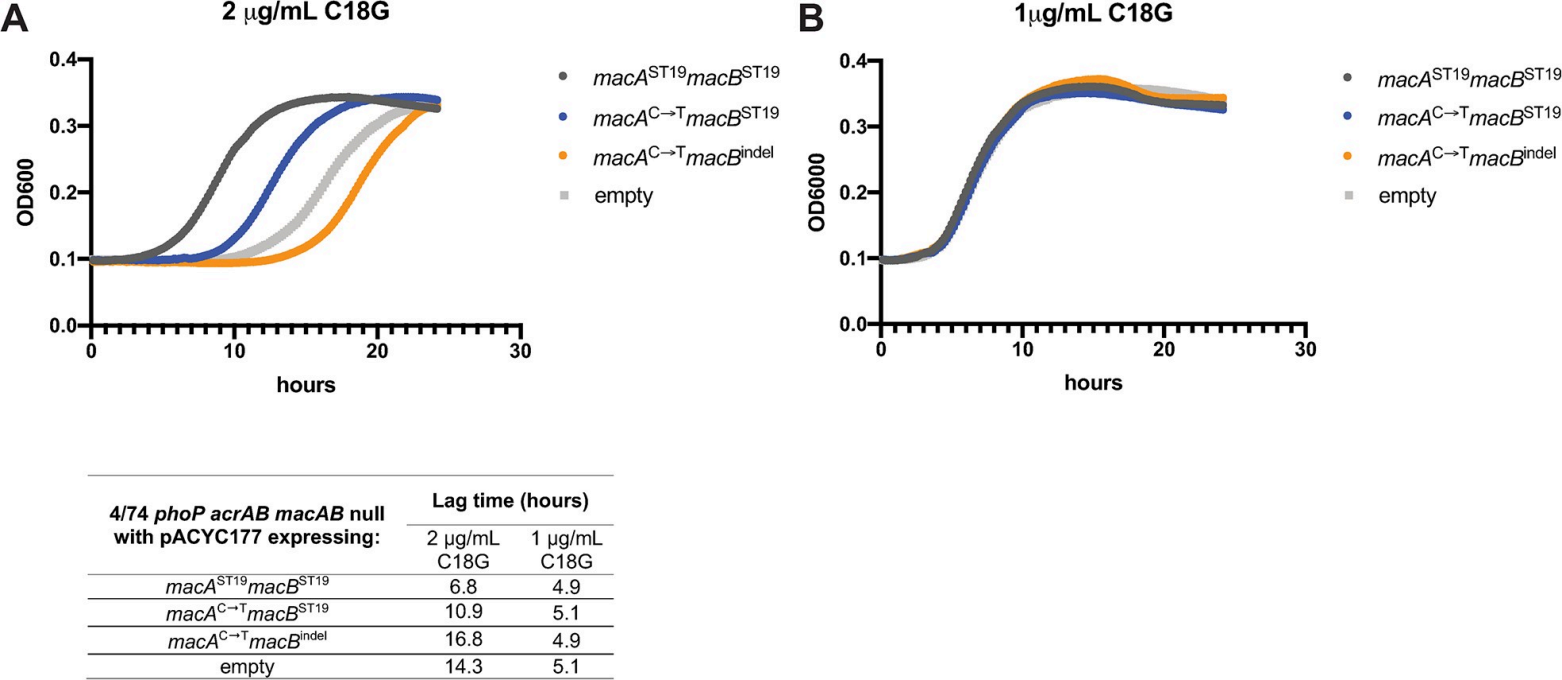
*S*. Typhimurium ST19 *macAB* provides superior resistance to the antimicrobial peptide C18G. 4/74 *phoP acrAB macAB* null mutants with low-copy pACYC177 plasmids constitutively-expressing *macAB* variants (pJH14-17, see S3 Table) were grown in N minimal medium, pH 7.4 and 1mM MgCl_2_. Overnight stationary phase cells were washed and normalized to OD600 = 1 before 1:200 final dilution into fresh N minimal medium with 2μg/mL C18G (A) or 1μg/mL C18G (B). OD600 was monitored over time using a BioTek Synergy HTX plate reader. Growth curves presented here are from one experimental run and representative of three independent experiments. Plotted data points are the geometric mean of quadruplicate or triplicate microplate wells. Lag time (inset table) was determined as time to reach OD600 = 0.15.

We took a reductive approach to quantify the impact of *macAB* genotype on antimicrobial peptide resistance. We reasoned that the contribution of MacAB to antimicrobial peptide resistance could be obscured *in vitro* by the profound PhoP-induced membrane modifications that dramatically slow antimicrobial peptide interactions with the cell envelope [59]. Furthermore, the constitutively expressed AcrAB-TolC pump effluxes a wide variety of compounds and can mask the contributions of other efflux pumps like MacAB during *in vitro* tests of antibiotic resistance [34,60]. Thus, we compared the contributions of MacAB variants to *Salmonella* growth in the presence of the antimicrobial peptide C18G using 4/74 *phoP acrAB macAB* null mutants harboring low-copy plasmids constitutively expressing *macA*^ST19^*macB*^ST19^ (the ST19 genotype), *macA*^C➝T^*macB*^ST19^ (the ST313 *macA* SNP alone), or *macA*^C➝T^*macB*^indel^ (the ST313 lineage 2 genotype). We found that the *macA*^ST19^*macB*^ST19^ plasmid facilitates growth of the 4/74 *phoP acrAB macAB* null mutant in minimal medium in the presence of 2μg/mL C18G (Fig 4A), while cells expressing *macA*^C➝T^*macB*^ST19^, *macA*^C➝T^*macB*^indel^, or carrying empty plasmids show much longer lag times (Fig 4A). With a subinhibitory level of C18G treatment (1μg/mL), all strains grew equally well, indicating the differences in lag time are not due to toxicity from expression of MacAB variants (Fig 4B). Comparison of lag times (Fig 4, inset table) suggests that the *macA*^C→T^ SNP impairs resistance to C18G while the *macB*^indel^ further disables the MacAB-TolC channel. These data demonstrate that the ST19 *macAB* genotype assists growth in the presence of inhibitory concentrations of C18G and can do so independently of other PhoP-induced genes.

### MacAB contributes to the ability of the *S*. Typhimurium ST19 isolate 4/74 to outcompete the iNTS ST313 isolate D23580 in the gut

**Fig 5:**
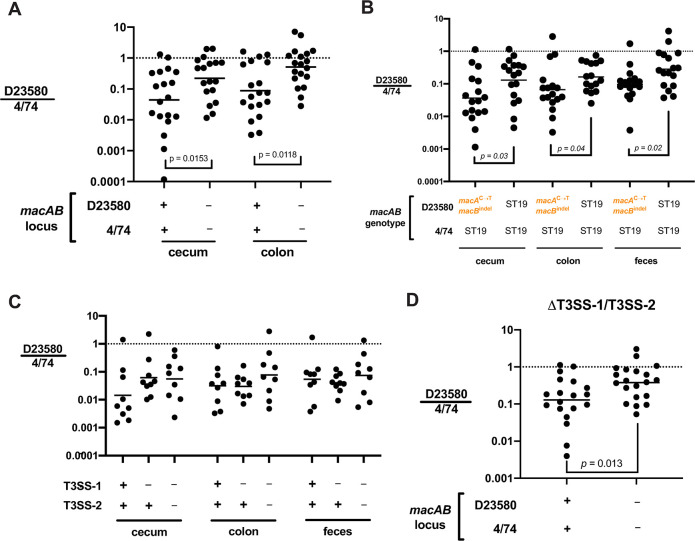
*macAB* influences fitness of *S*. Typhimurium D23580 in competition with 4/74 after oral infection. C57BL/6J mice (*Nramp1*^*-/-*^) were orally gavaged with streptomycin one day prior to oral infection with 5x10^7^ CFU each of a mixture of 4/74 and D23580 in PBS. Tissues were isolated for CFU determination and calculation of competitive index at day 2 after infection. (A) Competitive index of unmodified D23580 (“+”) versus unmodified 4/74 (“+”) compared to the competitive index of D23580 *macAB* null (“‒”) versus 4/74 *macAB* null (“‒”). (B) Competitive index values of 4/74 versus D23580, or 4/74 versus D23580 *macA*^ST19^*macB*^ST19^. Two-way repeated measures ANOVA on log-transformed data with Sidak’s post-test within organ, *p-*values as indicated in (A, B). (C) Competitive index of D23580 versus 4/74 and their T3SS-1 (*orgA* null) or T3SS-1/2 (*orgA ssaV* null) mutants. Two-way repeated measures ANOVA on log-transformed data with no significant differences in (C). (D) Competitive index of D23580 versus 4/74 T3SS-1/T3SS-2 mutants (*orgA ssaV* null) compared to competition of T3SS-1/2 mutants with full deletions of *macAB* (*orgA ssaV macAB* null) in each isolate. *t*-test on log-transformed data from the cecum in (D). Dots are competitive index values for individual mice with lines plotted at the geometric mean. See S1 Fig for CFU/g values.

Previous work has shown that MacAB promotes survival of *Salmonella* in the mouse gut after oral infection of C57BL/6 mice [38]. In addition, gut colonization is known to induce transcription of PhoP/Q regulated genes in *Salmonella* [61]. We thus hypothesized that the *macAB* mutations acquired by African *S*. Typhimurium ST313 isolates may impact fitness in the gut. To test this, we pretreated C57BL/6J mice with streptomycin and orally infected the next day with an equal mixture of 4/74 and the ST313 lineage 2 isolate D23580. We measured cecum and colon CFUs at day 2 after infection to calculate a competitive index (CI) and found that the ST19 strain 4/74 outcompeted D23580 by ~20-fold (Fig 5A). To test whether *macAB* contributed to this fitness difference, we competed *macAB* null mutants of 4/74 and D23580. The relative fitness of D23580 in gut tissues improved by approximately 5-fold when both isolates lacked *macAB* (Fig 5A), indicating that the ability of 4/74 to outcompete D23580 in the gut was partly *macAB*-dependent. To test whether the ST19 *macAB* genotype modulated D23580 fitness in the gut, we competed unmodified 4/74 with the D23580 *macA*^ST19^*macB*^ST19^ mutant. We found that altering *macAB* locus SNPs to the *macA*^ST19^*macB*^ST19^ genotype in D23580 improved its relative fitness in the cecum, colon, and feces when in competition with 4/74 (Fig 5B). These data show that the lower fitness of lineage 2 isolate D23580 in the mouse gut is partly due to its *macAB* genotype.

For *S*. Typhimurium, the type-3 secretion systems (T3SS) encoded by the SPI-1 and SPI-2 pathogenicity islands are important for inducing colitis during gut infection [62,63]. Previous work has shown *S*. Typhimurium ST313 isolates from Africa induce less host inflammation due to lower SPI-1-mediated invasion activity and reduced levels of flagellin expression when compared to ST19 strains [19,26,64]. Since *macAB* was shown to play a role in the streptomycin pre-treatment model of colitis [38], we suspected that 4/74 could be using SPI-1 and/or SPI-2-induced inflammation in the gut to outcompete D23580. Accordingly, we competed 4/74 and D23580 strains that lacked T3SS-1 (*orgA* null mutants) or T3SS-1/T3SS-2 (*orgA ssaV* null mutants). The competitive advantage of 4/74 over D23580 was not dependent upon the presence of T3SS-1 or T3SS-1/2 to induce gut inflammation (Fig 5C). However, there was a significant ~2.9-fold improvement in relative fitness of D23580 *orgA ssaV macAB* null versus 4/74 *orgA ssaV macAB* null mutants in the cecum compared to their *orgAssaV* null mutants with unmodified *macAB* (Fig 5D). These data demonstrate that *macAB* can influence relative fitness of D23580 versus 4/74 in the gut in the absence of T3SS-1/T3SS-2 induced inflammation.

### Host genetics shape the utility of *macAB* genotypes for D23580 systemic infection

**Fig 6:**
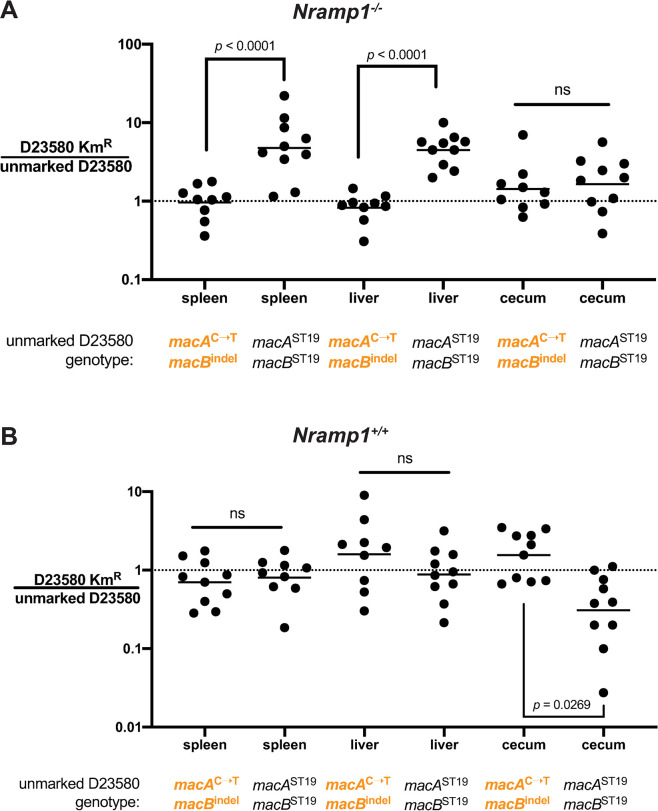
Host *Nramp1* genotype alters the utility of the D23580 *macAB* genotype during systemic infection. Isogenic competition of a kanamycin-marked D23580 with parent D23580 or D23580 *macA*^ST19^*macB*^ST19^ in (A) *Nramp1*^*-/-*^ C57BL/6J mice or (B) *Nramp1*^+/+^ C57BL/6J mice, day 3 post oral infection after streptomycin pre-treatment. Two-way ANOVA with repeated measures on log-transformed data using Sidak’s post-test within organ, *p* values as indicated. Dots are competitive index values for individual mice with lines plotted at the geometric mean. See S2 Fig for CFU/g values.

Since we saw no impact of the ST19 *macAB* genotype on D23580 replication in RAW264.7 cells, we predicted that ST313 *macAB*-associated SNPs might be dispensable or otherwise have no impact on the ability of D23580 to spread to systemic tissues. To exclude the contributions of known differences between ST19 and African ST313 in their inflammatory and disseminating behaviors *in vivo* [19,24,26,64], we competed D23580 with isogenic D23580 *macAB* mutants. To calculate a competitive index, we created a marked D23580 strain by placing a kanamycin resistance cassette at an intergenic site on the D23580 chromosome (see Materials and Methods) that was chosen for its low transcriptional activity when assessed by RNA-seq under a variety of growth conditions. After oral infection of C57BL/6J mice with the marked D23580-Kan^R^ strain and the parent D23580, we observed a competitive index of 1 in the gut and systemic sites, showing that the intergenic kanamycin marker insertion did not affect fitness in this model (Fig 6A). We found that the D23580 strain modified to the *macA*^ST19^*macB*^ST19^ genotype was outcompeted more than 4-fold by the isogenic D23580-Kan^R^ strain in systemic sites (liver and spleen), while showing no difference in the cecum (Fig 6A). This implies the *macA*^C➝T^*macB*^indel^ genotype provides an advantage to D23580 during systemic infection.

One important hypothesis regarding the emergence of African iNTS lineages is that the immune status of certain human populations in sub-Saharan Africa provides a permissive niche for systemic *S*. Typhimurium infection, thus uniquely shaping *Salmonella* evolution [15,65–67]. In our infection experiments with RAW264.7 macrophages (BALB/c origin) and C57BL/6J mice, the host gene *Nramp1* is not functional. BALB/c and C57BL/6J mice have a Glycine to Aspartic Acid (G169D) mutation in Nramp1 (i.e. a genotype of *Nramp1*^D169/D169^) which inactivates Nramp1 to yield a more permissive environment for *Salmonella* replication within macrophages [68]. Nramp1 dramatically restricts intracellular bacterial infection at systemic sites through the removal of magnesium and other divalent cations from the vacuolar environment [68–72]. However, Nramp1 has further influence on the host immune response. For example, *Nramp1*^+/+^ mice have more rapid innate responses than isogenic *Nramp1*^-/-^ mice, with higher levels of interferon γ (IFN-γ) and increased influx of neutrophils during the streptomycin pretreatment model of *Salmonella*-induced colitis [73] and in the dextran sodium sulfate (DSS)-induced colitis model [74]. Furthermore, *Nramp1*^+/+^ dendritic cells produce more inflammatory cytokines than *Nramp1*^-/-^ dendritic cells during *Salmonella* infection [75], an important route for the early, rapid dissemination of D23580 into the mesenteric lymph nodes [24]. These published data suggest that, in addition to the role of Nramp1 in control of intramacrophage replication of *Salmonella*, many relevant parameters of the immune response differ between *Nramp1*^-/-^ and *Nramp1*^+/+^ mice, especially in the amount of IFN-γ produced [76]. Furthermore, IFN-γ, in conjunction with other stimuli, induces maximal upregulation of *Nramp1* transcription [9]. Thus, *Nramp1* genotype has pleiotropic effects on the course of *Salmonella* infection. Given our results in a mouse background with defective Nramp1 (Fig 6A), we thus sought to test the effects of D23580 *macAB* genotypes during infection in mice with a more robust immune response.

We performed competitive oral infections after streptomycin pretreatment in resistant *Nramp1*^G169/G169^ C57BL/6J mice (hereafter, *Nramp1*^*+/+*^ mice). When we competed D23580-Kan^R^ with either the parent D23580 or D23580 *macA*^ST19^*macB*^ST19^ in *Nramp1*^*+/+*^ mice, systemic loads in liver and spleen at day 3 were equivalent, regardless of *macAB* genotype (Fig 6B). However, in this *Nramp1*^*+/+*^ host environment the *macA*^ST19^*macB*^ST19^ genotype did confer a fitness advantage to D23580 in the cecum (Fig 6B). This fitness advantage for the *macA*^ST19^*macB*^ST19^ genotype on the D23580 background is analogous to our observations in gut tissues when competing D23580 with 4/74 in C57BL/6J mice that are *Nramp1*^*-/-*^ (Fig 5B).

Overall, our murine infection and competition experiments show that *S*. Typhimurium ST313-associated *macAB* SNPs and indels influence *Salmonella* fitness depending, in part, on the host *Nramp1* genotype. During gut infection, the ST19 *macAB* genotype provides a fitness benefit to D23580 in competition with an ST19 isolate in permissive *Nramp1*^*-/-*^ mice (Fig 5). We also show that the impact of the ST313 lineage 2 *macAB* genotype upon systemic colonization by D23580 is Nramp1-dependent (Fig 6A).

## Discussion

We have provided new insights into the function of the MacAB-TolC channel in *Salmonella* Typhimurium pathogenesis by exploring the consequences of variations in the 5’-UTR and coding sequence of *macAB* that are found within ST313 lineages associated with invasive disease in Africa. We show that *macAB* expression is influenced by PhoP and that the ST19 *macAB* genotype improves functional resistance to the antimicrobial peptide C18G. ST313-associated variations in *macAB* genotype can affect antimicrobial peptide resistance as well as replication in a permissive macrophage-like cell line. In mice, the ST313-associated *macAB* gene variants contribute to the lower relative fitness of ST313 lineage 2 isolate D23580 in competition with the ST19 strain 4/74 in inflamed and uninflamed settings in the gut.

Previously, *macAB* mutants of *S*. Typhimurium were reported to show impaired oxidative stress resistance [38]. However, *S*. Typhimurium *macAB* null mutants of 4/74 did not show survival defects after peroxide treatment when compared to the parent strain (S3A Fig). Furthermore, sub-lethal concentrations of hydrogen peroxide did not induce transcription of *macAB* after short-term *in vitro* exposure (Fig 2A) or in time course assays with the 4/74 *macAB*::*lacZY* transcriptional reporter strain (S3B Fig). Poly-specificity allows efflux pumps to take on various functional roles since net cellular resistance to toxic compounds results from redundancies in efflux pumps and dynamic properties of the outer membrane [77]. Given the poly-specificity of efflux channels like MacAB-TolC, it is likely that, in addition to antimicrobial peptides, some bacterially-generated molecules are exported to directly quench reactive oxygen species (ROS), as proposed by Bogomolnaya and colleagues [38,78]. However, this oxidative stress resistance function of MacAB may depend upon experimental conditions and the bacterial genetic background.

We thus do not exclude a direct role for *macAB* in oxidative stress resistance in *Salmonella* but propose that an additional function is to counteract the effects of antimicrobial peptides encountered in both the gut and systemic sites. Indeed, bacterial responses to oxidative stress and antimicrobial peptide exposure are tightly linked by the PhoP/Q two-component regulatory system [79,80]. Antimicrobial peptide exposure induces transcription of genes in the RpoS regulon that assist bacterial adaptation to oxidative stress [81,82]. We have shown that *macAB* variants confer distinct levels of antimicrobial peptide resistance independently from other PhoP*-*mediated effects. Notably, on its own the *macA*^C➝T^ SNP which leads to the S_174_L mutation in MacA reduces the utility of MacAB in resistance to C18G (Fig 4). The role played by the PhoP/Q system in upregulating transcription of *macAB* is consistent with the MacAB-TolC channel of *Salmonella* countering antimicrobial peptide exposure, either through direct efflux or by assisting translocation of other bacterial factors important to cope with antimicrobial peptide stress.

Although one publication suggested PhoP repressed *macAB* transcription in *Salmonella* after low magnesium treatment [35], other published data suggest that *macAB* is positively regulated by PhoP. We have previously noted that *phoPQ* null mutants have lower expression of *macAB* when grown in InSPI2 medium [50], while others have seen that *S*. Typhi production of MacA protein under low magnesium conditions is PhoP-dependent [83]. Furthermore, *macAB* is important for *S*. Typhimurium replication in macrophages [38,56], a niche where PhoP/Q signaling is active and important for *Salmonella* survival [55,61]. In addition to the different *Salmonella* isolates used across studies, our results suggesting PhoP promotes *macAB* transcription may arise from differences in experimental conditions, the importance of regulatory kinetics such as PhoP turnover [38], changes in ribosomal RNA levels in response to low magnesium [84], or coordinated activity with other transcriptional regulators [59]. We conclude that PhoP-inducing signals generally serve to maximize *macAB* transcription in *Salmonella*.

The *macAB* locus contains additional regulatory complexity that requires further investigation. While we show that the 5’-UTR_*macA*_^Lin2.1^ SNP is sufficient to impair replication of 4/74 in macrophages (Fig 3A), the mutation did not affect the level of *macAB* transcription in low magnesium medium (S4 Fig). The mapped transcriptional start site (TSS) of *macA* is 504 nucleotides upstream of the start codon and is located within the divergently-transcribed gene *ybjX* (also known as *somA*) (see Fig 1B and https://tinyurl.com/macAB-Jbrowse). Long 5’-UTRs have been shown to have trans-acting regulatory roles in expression of virulence genes in both *S*. Typhimurium [43,85] and *S*. Typhi [42]. Such 5’-UTR elements can post-transcriptionally regulate gene expression through formation of RNA secondary structures that control interactions with small regulatory RNAs, or by directly influencing transcript stability [86]. The 5’-UTR_*macA*_^Lin2.1^ SNP in particular is near the ribosome binding site of the *macA* transcript which could impact translation initiation (see Fig 1B). We observed that expression of MacAB from a high-copy plasmid (pUC19) or a plasmid with an arabinose-inducible promoter is toxic to cells, a lethality phenomenon reported by others [29]. Additionally, MacA exhibits high binding affinity for TolC *in vitro*, suggesting it may outcompete other TolC-interacting efflux pumps whenever MacAB is expressed [87]. Taken together, these observations support the conclusion that the tight transcriptional regulation of *macAB* functions to provide utility without compromising other cellular functions.

We have not demonstrated the direct structural consequences for the MacAB-TolC channel that result from the *macAB* SNPs present in ST313 isolates. Such changes might include assembly kinetics of the MacA hexamer, stability or mechanical function of the tripartite complex, or perhaps altered association with other important constituents of the Gram-negative cell envelope [37]. The *E*. *coli* MacA protein can bind tightly to rough LPS via residues on the N-terminus with affinity that is higher than that of polymyxin B, prompting the suggestion that rough LPS may be a cargo of the channel [29]. Given that PhoP is required for maximal *macAB* transcription and that MacAB-mediated resistance to C18G did not require other PhoP*-*regulated genes (Fig 2B and 2C), we speculate that rough LPS-binding by MacA is a structural feature important for its antimicrobial peptide resistance function. While the ST313 *macA*^C➝T^ SNP resulting in the S_174_L mutation impairs MacAB-mediated resistance to C18G, it is possible this SNP variant improves efflux of other substrates not tested here and thus might serve a direct role in the pathogenic adaptation of African iNTS. Further structural investigation of the *Salmonella* MacAB-TolC channel will provide new insights into the role of this important efflux pump in *Salmonella* physiology and host-pathogen interactions.

It is not immediately clear why *S*. Typhimurium ST313-associated *macAB* gene variants lead to different infection outcomes depending on strain background; however, some possibilities may be proposed based on current knowledge. In the case of D23580 and other ST313 lineage 2 isolates, the mutation responsible for the marked increase in expression of the PhoP-regulated protease PgtE [88] could sufficiently compensate for antimicrobial peptide resistance otherwise provided by the ST19 *macAB* genotype. In this case, the ATP-dependent MacAB-TolC channel might siphon energy or even directly interfere with other genes or behaviors unique to the D23580 strain background. Such loss-of-function mutations that favor the activity of other pathogenesis mechanisms have been noted in *Salmonella* evolution. For *Salmonella* Typhi, a stop codon that interrupts *fepE* prevents very-long O-antigen production [89], which in turn permits the horizontally-acquired Vi capsule to have maximal immune evasive effects during systemic infection [90]. Finally, it has been shown that point mutations in the highly expressed AcrAB-TolC efflux pump can alter expression of a variety of *Salmonella* pathogenic genes indirectly without affecting expression of other efflux pumps [91]. Further research will be required to identify other *Salmonella* genes that might interact with the *macAB* genotype to influence pathogenesis.

In single infections, *S*. Typhimurium D23580 and other ST313 isolates readily colonize the intestinal tracts of a variety of animals including primates, rodents, and chickens [23,24,92–94]. A recent study of competitive fitness in the streptomycin-pretreatment model of mouse colitis showed that D23580 can outcompete the ST19 isolate IR715, a strain that is derived from the common laboratory reference isolate 14028S [23], further confirming the ability of ST313 to infect multiple animal species. We show that differences in *macAB* genotype are partly responsible for a relative fitness defect of D23580 during competition with the ST19 isolate 4/74 in the inflamed and uninflamed gut (Fig 5A, 5B and 5E). Several factors could contribute to these differences in competitive fitness of D23580 versus certain ST19 strains. Both D23580 and IR715 do not express *sopE*, a SopEΦ prophage-encoded virulence factor delivered by the T3SS of SPI-1 [19,26]. In contrast, *sopE* is present in 4/74 and the closely-related ST19 isolate SL1344 [95]. *sopE* is known to drive substantial cecal inflammation in the streptomycin pretreatment model of colitis [96]. Further, the ST19 strains SL1344 and 4/74 contain the pCol1b plasmid and can produce colicin Ib, potentially helping *Salmonella* outcompete other *Enterobacteriaceae* in the inflamed gut [97]. We found that ST19-associated *macAB* gene variants increase fitness of D23580 in scenarios with robust gut inflammation, such as in competition with a *S*. Typhimurium ST19 isolate that has *sopE* [96] and in mice with *Nramp1* functionality [73]. However, when D23580 infects a more permissive host, such as *Nramp1*^-/-^ C57BL/6J mice, its ST313 lineage 2 *macAB* variant is advantageous for systemic infection (Fig 6B). Thus, D23580 shows a degree of specialization for systemic infection that depends upon both its *macAB* genotype and parameters of the host innate immune response. Our experiments reinforce the value of testing *Salmonella* isolates in various host genotypes to identify potentially important host-pathogen gene interactions.

Although no studies have specifically linked *NRAMP1/SLC11A1* polymorphisms to the susceptibility of humans to invasive *Salmonella*, several *NRAMP1* polymorphisms found within West African populations are strongly linked to susceptibility to intracellular infection with *Mycobacterium tuberculosis* [98,99]. We note that numerous parameters in mammalian hosts that are related to susceptibility to invasive *Salmonella* disease are also compromised in *Nramp1-*deficient mice, as summarized below. For example, macaques that were previously infected with simian immunodeficiency virus have diminished IL-17 production that reduces the influx of neutrophils into the gut, thus compromising control of *Salmonella* dissemination from the gut [100]; similarly, neutrophil influx into the gut during *Salmonella* colitis is delayed in *Nramp1*^*-/-*^ mice [73]. Nramp1 also regulates the oxidative burst capacity of phagocytes [101]. A reduced oxidative burst response is observed in Gambian children recovering from malaria infection [102] and is likely a consequence of malaria-induced hemolysis as revealed by experiments in mice [65].

In humans, *STAT4* variants have been linked to increased risk for invasive *Salmonella* infection. Specifically, a genome-wide-association study identified a *STAT4* polymorphism in children in Kenya and Malawi which correlated with reduced IFN-γ responses of *ex vivo* stimulated immune cells [103]. The importance of IFN-γ to control of *Salmonella* infection at both acute and chronic stages has been clearly demonstrated [104], and upregulation of *NRAMP1* in macrophages is part of the response to IFN-γ [105]. Malaria, malnutrition, and HIV can compromise innate resistance to intracellular bacterial infection through a wide variety of mechanisms, including alterations in macrophage responsiveness to IFN-γ [106]. Based on outcomes of our competitive infections using *Nramp1*^*-/-*^ and *Nramp1*^*+/+*^ mice, we speculate that the *S*. Typhimurium ST313-associated *macAB* gene variants represent adaptive evolution for systemic infection when restriction of *Salmonella* dissemination is compromised in the host.

Oral infection of mice with *Salmonella* Typhimurium after streptomycin pretreatment causes substantial inflammation and colitis, which depends on the function of SPI1 and SPI2-encoded type three secretion systems [107]. Although D23580 exhibits less T3SS-1 mediated invasion *in vitro* and in animal infections [19,26], we found that D23580 was outcompeted by 4/74 in the mouse gut whether or not both isolates lacked T3SS-1 or T3SS-1/2, and this fitness defect was partly *macAB*-dependent (Fig 5D and 5E). Although all of these experimental models vary in degree and type of inflammation, they share a diverse and dynamic set of host- and microbe-generated antimicrobial peptides. We suggest that MacAB contributes to *Salmonella* pathogenesis by countering antimicrobial peptide stress as part of the bacterial response orchestrated by the PhoP/Q two-component system.

The *S*. Typhimurium ST313-associated *macAB* variants we have characterized suggest a pattern of evolutionary convergence toward a degraded function of the MacAB-TolC efflux pump. While MacAB remains functional amongst *S*. Typhimurium lineages associated with gastroenteritis, we conclude that inactivation of the MacAB system within African ST313 lineages represents a unique adaptation that may facilitate systemic infection of permissive hosts.

## Materials and methods

### Vertebrate animal ethics statement

All animal experiments were approved by the Stanford University Administrative Panel on Laboratory Animal Care (APLAC) with oversight by the Institutional Animal Care and Use Committee (IACUC) under local Protocol ID 12826. Animals were housed at specified-pathogen free (SPF) level in University facilities accredited by the Association of Assessment and Accreditation of Laboratory Animal Care (AAALAC) International.

### Phylogenetic tree construction and *macAB* status visualization

The sources of all the genomic data files used are listed in S1 Table. The assembled genomes of 18 *S*. Typhimurium strains, including the ST19 representative strain 4/74 [108] and ST313 representative strain D23580 [9], were obtained from GenBank, while the raw sequencing data of 267 *S*. Typhimurium ST313 strains derived from previous publications [12,13,15,16,109] were downloaded from SRA (https://www.ncbi.nlm.nih.gov/sra/) and EMBL-EBI (https://www.ebi.ac.uk/) databases. To root the phylogenetic tree, the genome of *Salmonella* Typhi CT18 was downloaded from GenBank and used as the outgroup.

SNIPPY v4.4.0 [110] was used to map the sequencing data against 4/74, call the SNPs, construct pseudo-genomes, and make a genome alignment. SNIPPY used Freebayes [111] as the variant caller, the default parameter of minimal coverage was 10, and the minimal fraction was 0. For the assemblies from GenBank, SNIPPY used contigs as the input. Recombinant regions were detected and removed from the alignment using Gubbins v2.4.1 [112]. RAxML-NG v0.9.0 [113] was used to build a phylogenetic tree, with substitution model GRT+G. The phylogenetic tree was visualized on iTol [114] (https://itol.embl.de/). Based on the phylogeny and prior publications, the ST313 strains were classified into UK ST313 [15], Brazil ST313 [16], lineage 1 [12], lineage 2 [12], lineage 2.1 [13], and lineage 2.2 [109].

The *macA* 5'-UTR, *macA*, and *macB* sequences of strain 4/74 were used to generate BLAST databases with BLAST 2.9.0+ [115]. Raw reads of all the strains were assembled using Unicycler v0.4.8 [116]. The quality of assembly was checked by Quast v5.0.2 [117]. The N50 value of all assemblies was >20kb, and the number of contigs was <600. The genome assemblies were queried against the databases using the BLASTn algorithm.

### Bacterial strains, plasmids, primers and growth conditions

See S2 Table for bacterial strains used in this study. Where applicable, P22 *HT105/1 int-201* phage was used to move marked mutations from previously-generated *S*. Typhimurium mutants into the 4/74 strain according to standard protocols. *Salmonella* were routinely grown in LB Lennox (10g/L tryptone, 5g/L yeast extract, 5g/L NaCl) for cloning manipulations or in LB Miller (10g/L NaCl) for infection assays with the following antibiotic concentrations: streptomycin, 200μg/mL; chloramphenicol, 25μg/mL; tetracycline, 15μg/mL; kanamycin, 40μg/mL; and gentamicin, 25μg/mL (GoldBio).

See S3 Table for plasmids and S4 Table for primers used in this study.

### InSPI2 and intramacrophage RNA-seq data

RNA-seq transcript per million (TPM) values were extracted from previously published SalCom datasets [18,36,49] and used to calculate fold-change expression values presented in Fig 2.

### Lambda (λ) red recombination for marked mutants

Marked mutants were made based on the λ red procedure [118] using the temperature sensitive pSIM5-Tet plasmid that contains a temperature shock inducible promoter driving recombinase expression [20]. Briefly, primers with 40bp homology targeting the flanking regions of gene to be deleted were used to amplify the FRT-flanked kanamycin cassette from pKD4. 4/74 or D23580 / pSIM5-Tet cells were grown with streptomycin and tetracycline at 30°C overnight in LB Lennox before subculturing 1:100 into fresh mediumand growing to an OD of 0.4. The culture was then incubated at 42°C shaking for 15 minutes before placing on ice make cells competent for electroporation. Cells were washed with chilled double-distilled water, followed by electroporation of 800ng of purified PCR product. Cells were recovered in SOC for 1.5 hours shaking at 30°C before pelleting and plating on LB kanamycin plates. After overnight growth at 37°C, individual colonies were struck across fresh plates to purify single colonies, followed by colony PCR to confirm correct insertion of the kanamycin cassette. For 4/74, marked mutants were moved into a clean 4/74 strain background using P22 *HT105/1 int-201* phage, and the correct insertion was confirmed by PCR.

For construction of D23580-Kan^R^ we amplified the kanamycin resistance cassette from pKD4 using primers del_23_F and del_23_R for insertion by λ red recombination as described above. These primers target the Kan^R^ cassette to the intergenic region between *STMMW_41451* and *STMMW_41461* between coordinates 4441510 and 4441511 in D23580 (GenBank: FN424405.1). This region is part of the remnant prophage Def4 [20] and not transcribed in RNA-seq datasets in variety of *in vitro* conditions [18]. Correct insertion of the KanR cassette was confirmed by primers Fw_STM4196-7_ext and Rv_STM4196-7_ext. D23580-Kan^R^ was confirmed to be cured of temperature sensitive pSIM5-Tet after growth at 37°C by testing isolated colonies for tetracycline sensitivity.

### Construction of *lacZY* reporter strains

Chromosomal β-galactosidase (*lacZY*) transcriptional fusions were made using the method of Ellermeier and colleagues [119]. 4/74 marked mutants with FRT-flanked kanamycin cassettes derived from pKD4 were electroporated with 200ng pCP20, recovered for 1 hour in SOC at 30°C before plating dilutions on LB streptomycin chloramphenicol plates, followed by growth at 30°C overnight. Colonies were re-struck to purify for single clones, then patched onto LB kanamycin and LB streptomycin chloramphenicol plates to confirm loss of kanamycin by FLP-recombinase activity. Two kanamycin sensitive colonies were picked and grown in LB Lennox at 30°C, followed by electroporation with 200ng pCE36 plasmid purified from the *Salmonella* pir+ strain JS198/pCE36 [119]. Cells were recovered in SOC at 37°C and plated on LB kanamycin to select for pCE36 integration. Purified colonies were screened by PCR using P1 and Lac primers for integration of pCE36 into the FRT site [119].

### Scarless mutant generation

In order to study the influence of these nucleotide changes on *Salmonella* pathogenesis without otherwise altering *macAB* regulation or introducing other changes in the genome, we used scarless mutagenesis to make nucleotide changes in the genome of a given *Salmonella* isolate. We used the suicide plasmid pEMG system described by Martínez-García and de Lorenzo [120] and as applied to *Salmonella* Typhimurium by Owen and colleagues [20]. Genomic DNA from 4/74 or D23580 was used as template for Phusion (ThermoFisher) PCR of 1600bp flanking the polymorphism to be transferred. For marker-less deletions, 800bp flanking each side of the region to be deleted were amplified to generate two PCR products. Primers were designed to include ~20nt overhangs to permit Gibson Assembly of a single 1600bp fragment (for marker-less nucleotide changes) or the two 800bp flanking regions (for marker-less deletions) into pEMG that was previously digested with XbaI and KpnI-HF (New England Biolabs, NEB). Purified PCR product (Qiagen) and digested pEMG backbone were assembled with the DNA HiFi Assembly MasterMix (NEB). The 5’-UTR_*macA*_^Lin2.1^ SNP was incorporated into pEMG::*macA*^ST19^ or pEMG::*macA*^C➝T^ by PCR using primer pairs 1309,1310 and 1311,1312; the two PCR products were assembled with HiFi DNA Assembly MasterMix with pEMG previously digested with XbaI and KpnI-HF. A given pEMG::X plasmid was mobilized from *E*. *coli* S17-1 λpir into recipient *Salmonella* by conjugation, followed by selection for transconjugants on M9 minimal agar plates formulated with 0.2% glucose, 1mM MgSO_4_, and 40μg/mL kanamycin. Merodiploid transconjugants were resolved by electroporation of pSW-2 as previously described [20], followed by colony PCR and standard Sanger sequencing of PCR product to identify clones with the intended point mutations. Confirmed mutants were cured of unstable pSW-2 by several passages in LB before patch plating to confirm loss of pSW-2 by gentamicin sensitivity.

To confirm that key strains contained the intended engineered nucleotide(s) and no unintended mutations in other parts of the genome, the four strains 4/74 *macA*^C➝T^, 4/74 *macA*^C➝T^*macB*^indel^, D23580 *macA*^C➝T^*macB*^ST19^ and D23580 *macA*^ST19^*macB*^ST19^ were genome-sequenced using Illumina paired-end sequencing (SNPsaurus, Oregon) as indicated in S2 Table and aligned to reference genomes (NCBI) using CLC Genomics Workbench (Qiagen).

### Plasmid construction

The *macAB* sequence was amplified from genomic DNA templates with Phusion polymerase (ThermoFisher) using primers 1223 and 1224. The PCR product and pBAD33.1 (Addgene #36267) were digested with NdeI and HindIII-HF (New England Biolabs, NEB) followed by T4 ligation and transformation into NEB 10beta competent cells. The cassette inclusive of the T7 ribosome binding site, *macAB*, and the two transcriptional terminators was amplified with Phusion polymerase using primers 1272 and 1273 from pBAD33.1-*macAB* templates. The PCR products and destination pACYC177 were digested with ScaI and PstI, purified using the PCR purification kit (Qiagen), and ligated with T4 polymerase before transformation into NEB 10beta competent cells, selecting on kanamycin. The promoter for the *Amp*^*R*^ gene thus drives expression of the *macAB* gene inserted at the ScaI site within the *Amp*^*R*^ sequence of pACYC177.

### Stimulation conditions for PhoP-regulated gene expression

The procedures here are based on methods described by Bader and colleagues [52]. For low-magnesium treatment, cells were grown at 37°C overnight in N minimal medium with 100mM Tris-HCl pH 7.4, 0.2% deferrated Casamino acids (Chelex treated), 0.2% glycerol, and 10mM MgCl_2_ (high magnesium) with relevant antibiotics. Cells were subcultured 1:100 into fresh medium and grown to OD600 ~0.2, or about 4 hours. Cells were washed 3x in the same medium or medium with 10μM MgCl_2_ (low magnesium) and returned to the incubator, growing for a further 90 minutes. Where applicable, N minimal medium was buffered with 10mM MES at pH 5.8 or 4.9. For β-galactosidase assays, 1 mL of cell culture was then transferred to a microcentrifuge tube and placed on ice, followed by one wash in 1mL pre-chilled 100mM phosphate buffer, pH 7. A 150μL aliquot was removed and the OD600 was checked on a microplate reader (Synergy HTX, BioTek) and recorded. The aliquot was returned to the original tube and cells were pelleted again in a chilled centrifuge. The supernatant was removed and the pellet was frozen immediately on dry ice and transferred to -80°C storage.

For C18G stimulation, cells were grown overnight in N minimal medium, pH 7.4 (see above) formulated with 1mM MgCl_2_. We titrated C18G (Anaspec) to confirm a concentration that did not inhibit growth, identifying 5μg/mL as optimal, similar to what was reported by Bader and colleagues [52]. Cells were subcultured 1:100 and grown to OD ~0.2 before pelleting and resuspending in fresh media with or without 5μg/mL C18G. Cells were grown for a further 90 minutes and processed as described above.

### β-galactosidase assays

We used a modified lysis protocol and a kinetic microplate assay to measure β-galactosidase activity based on the methods described by Schaefer and colleagues [121] and Thibodeau and colleagues [122]. Bacterial pellets were removed from -80°C to room temperature, thawed briefly, then resuspended in 200μL of 100mM phosphate buffer, pH 7.0. A 200μL mixture of 20% PopCulture (Millipore) and 8U/μL of rLysozyme (Millipore #71110–4, Lot 3277983, 30U/μL) was added to each sample and vortexed for 5 seconds. After 5 minutes at room temperature, the sample was vortexed again and incubated for a further 5 minutes. During lysis optimization tests, this freeze/thaw and lysis buffer treatment achieved a >95% reduction in OD405 within two minutes, and the suspension was visually clear within 20 seconds. In each well of a 96 well microplate, 70μL of Z-buffer with 0.05M β-mercaptoethanol was added. After lysis, 80μL of sample was added in quadruplicate wells, with control wells having lysis buffer only; a lysed pellet of wild-type 4/74 cells with no *lacZY* reporter served as an additional control. Using a multichannel pipette, 30μL of ONPG (Sigma) previously dissolved in 100mM phosphate buffer at 4μg/mL was added to each well. The microplate was transferred immediately to a Synergy HTX (BioTek) plate reader that was pre-equilibrated to 28°C. OD420 was measured every minute for 90 minutes, incubating at 28°C with shaking between reads. The change in absorbance at 420nm over time was calculated from the slope of the reads and used in the standard Miller unit calculation,

$$Miller\;units=\frac{slope({OD}_{420})*1000}{{OD}_{600}*2.5*0.08}$$

where OD600 is the density of cells in 1mL before pelleting and freezing; 2.5 corrects for the pellet resuspension in a 400μL lysis volume; and 0.08 is the volume of sample (in mL) assayed per well.

### Antimicrobial peptide sensitivity assay

4/74 *phoP acrAB macAB* null mutants with indicated pACYC177 plasmids were grown aerobically overnight in N minimal media, pH 7.4 (100mM Tris-HCl) with 1mM MgCl_2_, 0.2% Casamino acids, and 0.2% glycerol with appropriate antibiotics. Cells were normalized to OD600 of 1 = 1x10^9^ cells/mL, then diluted 1:100 into fresh medium without antibiotics. 75μL of fresh medium alone or 2x final concentration of C18G (Anaspec, Fremont, CA) in fresh medium were added to wells of a sterile, polypropylene 96-well flat-bottomed plate (Griener BioOne, Product 655261). 75μL (~7.5x10^4^ cells) of the 1:100 bacterial suspension was added to wells in quadruplicate for a final 1:200 dilution of cells, with final μg/mL C18G as indicated. C18G was titrated previously, with higher concentrations (≥3μg/mL) preventing growth of all strains while treatment at concentrations ≤1.5μg/mL showing no differences in growth kinetics compared to untreated wells. The plate was sealed with a Breathe-Easy gas permeable film (Diversified Biotech) and OD600 was measured every ten minutes while growing at 37°C with linear shaking using a Synergy HTX plate reader (BioTek). Lag time was calculated as the time to reach OD600 = 0.150.

### Macrophage infection assays

RAW264.7 cells were passaged in DMEM + 10% FBS with 4.5g/L glucose and 110mg/L sodium pyruvate and L-glutamine. For infection, cells were seeded at 2.5x10^4^ cells per well in a 96 well plate starting 24 hours prior to infection. Single *Salmonella* colonies were picked from recently struck LB plates and grown overnight in LB Miller with appropriate antibiotics. After 16–18 hours of culture, cells were pelleted, washed once in PBS, and concentration was determined by OD600 of 1 = 1x10^9^ CFU/mL. Bacteria were diluted to 1x10^8^/mL and added to DMEM complete medium for a final 2.5x10^6^ CFU/mL concentration. Medium was aspirated from the RAW cells with a multichannel and replaced with 50μL of fresh media. 100μL of each inoculum (2.5x10^5^ CFU, MOI = 10) was added in quadruplicate for each time point (0, 8 and 20 hours). Plates were spun at 300g for 10 minutes at room temperature (21–25°C), then incubated at 37°C with 5% CO_2_ for 30 minutes to allow phagocytosis. Meanwhile, the inoculum was diluted 10-fold in PBS and plated to confirm the MOI. After 30 minutes, the medium was then aspirated and RAW cells were gently washed twice with 100μL medium, replacing finally with 100μL of DMEM complete with 100μg/mL gentamicin. The RAW cells were incubated at 37°C for 60 minutes, then the high gentamicin medium was removed and replaced with medium with 20μg/mL gentamicin to suppress extracellular growth of *Salmonella* for the remainder of the culture period (this was *t =* 0). At each timepoint, medium was aspirated and RAW cells were washed 3x with 150 μL PBS before lysis in 30μL of 1% Triton X-100 at room temperature. After 5 minutes, the wells were pipetted vigorously with a multichannel, then each well was topped with 120μL of PBS. The well suspensions were mixed, serially diluted in PBS, and spot-plated to calculate CFUs per well.

### Mouse strains

C57BL/6J (Stock Number 000664), mice were purchased from Jackson Laboratories. C57BL/6J *Nramp1*^G169/G169^ mice were previously described [123].

### Streptomycin pre-treatment model of colitis

Mice 7–12 weeks old were deprived of food briefly for 4 hours prior to gavage with 20mg streptomycin in sterile water. The next day, overnight stationary phase cultures of *Salmonella* strains grown with appropriate antibiotics were pelleted and washed twice in PBS before cell quantification by OD600. Twenty hours after streptomycin treatment, mice were again briefly deprived of food for 4 hours, followed by gavage of an equal mixture in PBS of 5x10^7^ CFU of each strain for a total inoculum of 1x10^8^ CFU. The inoculum was diluted and plated onto LB agar plates with appropriate antibiotics to differentiate strains and calculate the input ratio. Mice were euthanized by CO_2_ asphyxiation at a given timepoint, tissues were removed and homogenized in sterile PBS, then diluted and plated onto LB agar plates with different antibiotics to distinguish *Salmonella* strains. Total *Salmonella* CFUs were determined from streptomycin plates, while the D23580 proportion was calculated from streptomycin and chloramphenicol plates. In the isogenic D23580 competition, the addition of kanamycin identified the proportion of D23580-Kan^R^ colonies from the total D23580 population. The output ratio in tissues was divided by the input ratio of the inoculum to compute the competitive index.

### Statistical analysis

Data were analyzed with GraphPad Prism 8 (GraphPad Software, LLC). Statistical tests were performed as indicated in the figure legends.

## Supporting information

S1 Fig CFU counts corresponding to Competitive Index (CI) values of cecal tissue samples plotted in Fig 5. Fig S1A-D correspond to Fig 5A–5D, respectively. Connecting lines show paired values of CFU per gram from the cecum of an individual mouse.
(TIF)

S2 Fig CFU counts corresponding to Competitive Index (CI) values of spleen and cecal tissue samples plotted in Fig 6. Fig S2A-B correspond to Fig 6A and 6B, respectively. Connecting lines show paired values of CFU per gram from the spleen or cecum of an individual mouse.
(TIF)

S3 Fig *macAB* does not assist *in vitro* peroxide resistance in the ST19 strain 4/74. (A) Survival of 4/74 *macAB* null mutant after peroxide treatment. 4/74, 4/74 *macAB* null and 4/74 *rpoS*::Kan were grown in LB Miller overnight with appropriate antibiotics, normalized to OD600 = 1 before 1:100 dilution into fresh LB with or without 1mM H_2_O_2_, growing at 37°C while rotating. Cells were removed hourly and serial dilutions plated to calculate percent survival in reference to CFUs at *t =* 0. (B) Transcriptional induction after peroxide exposure. 4/74 parent and the 4/74 *macAB*::pCE36 *lacZY* transcriptional fusion were normalized to OD600 = 1 after overnight culture in LB, followed by 1:100 dilution into fresh LB medium and growth while shaking at 37°C. At OD600 = 0.5 (~2 hours of growth), mid-exponential cells were pelleted and resuspended in the same volume of fresh LB with or without 0.5mM H_2_O_2_. Cells were removed every 30 minutes and assayed for β-galactosidase production as described in Materials and Methods.
(TIF)

S4 Fig 5’-UTR_*macA*_^Lin2.1^ SNP does not alter transcriptional response of *macAB* to low Mg^2+^. Two clones of 4/74 *macAB*::pCE36 transcriptional fusion strains with the 5’-UTR_*macA*_^Lin2.1^ SNP preceding *macA* were grown to mid-exponential phase in N minimal medium pH 7.4 with high Mg^2+^ (10mM) then shifted to the same or low Mg^2+^ (10μM) media and grown for 90 minutes. β-galactosidase activity was measured using a kinetic Miller assay as described in Materials and Methods. The 5’-UTR_*macA*_^Lin2.1^ SNP was incorporated by λ red recombination using the primer pair 1288b, 1289 to amplify the Km^R^ cassette from pKD4. Transcriptional fusions generated with pCE36 include an internal, independent ribosome binding site for translation of *lacZY* from the transcript.
(TIF)

S1 Table Sources of genomic data. (XLSX)

S2 Table Bacterial strains. (PDF)

S3 Table Plasmids. (PDF)

S4 Table Primers. (PDF)
