# Supplementary figures and images for: Genetic variation in the MacAB-TolC efflux pump influences pathogenesis of invasive Salmonella isolates from Africa
Source: PLoS Pathog. 2020 Aug 24;16(8):e1008763. doi: 10.1371/journal.ppat.1008763 (PMC7446830; doi:10.1371/journal.ppat.1008763)

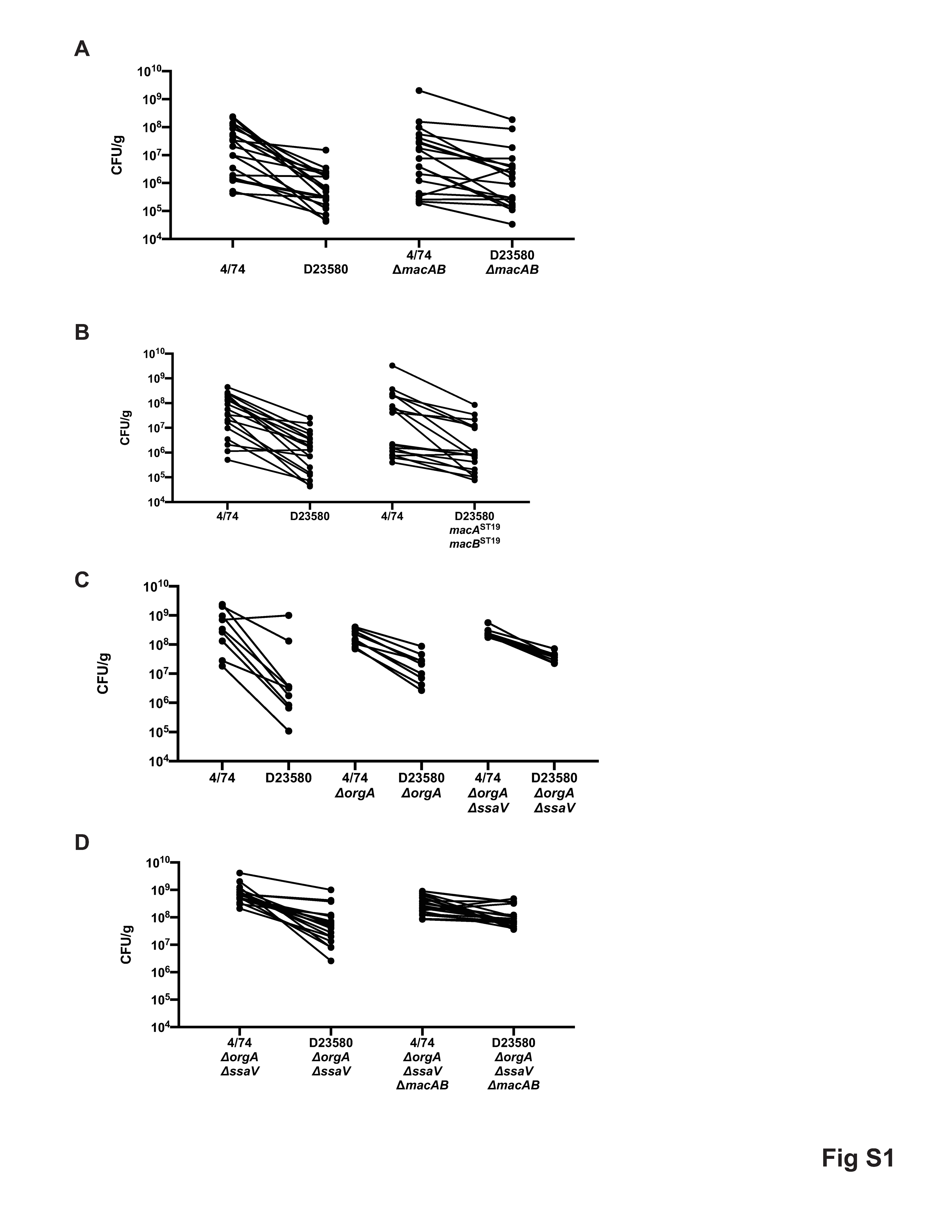

Supplement: S1 Fig — Fig S1A-D correspond to Fig 5A–5D, respectively. Connecting lines show paired values of CFU per gram from the cecum of an individual mouse. (TIF) [file ppat.1008763.s001.tif]

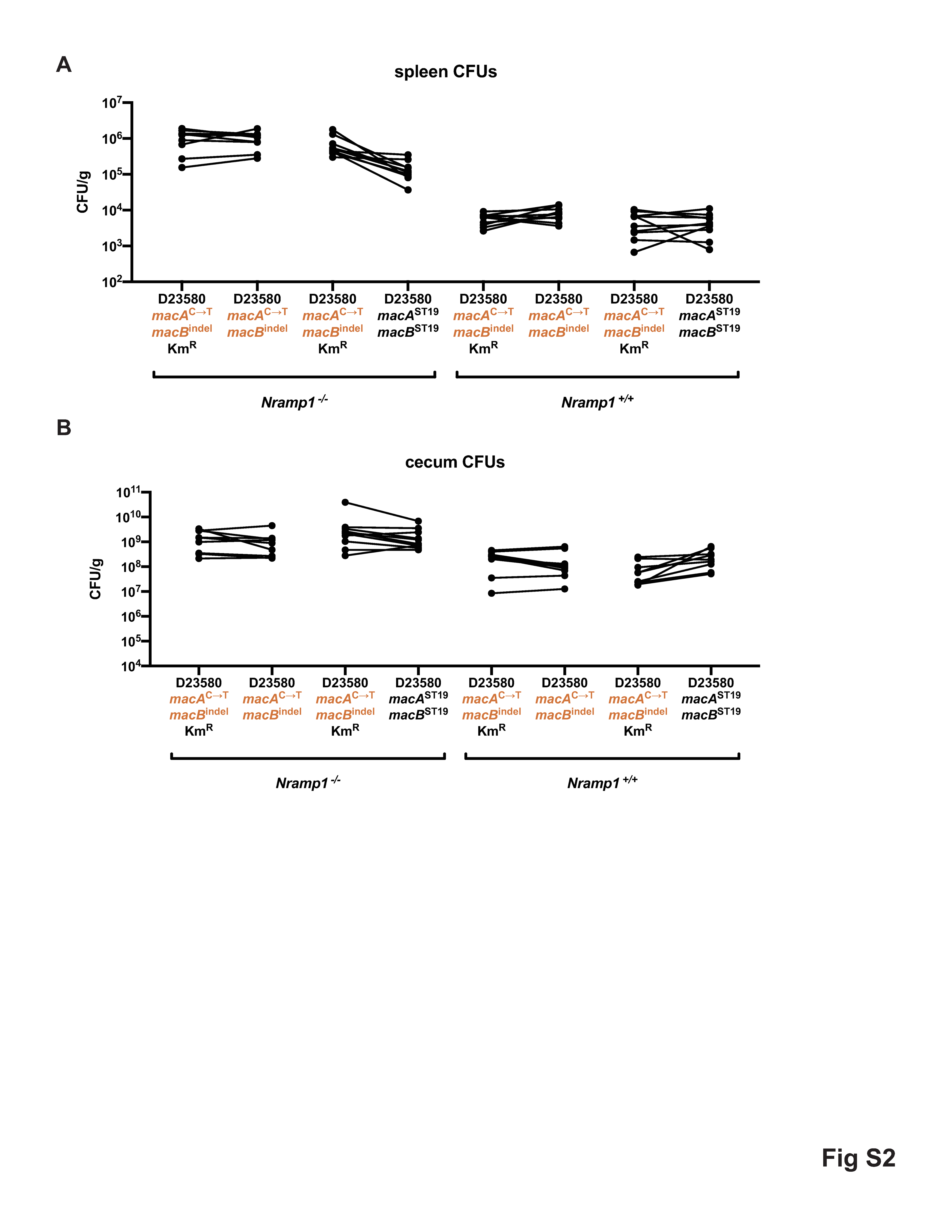

Supplement: S2 Fig — Fig S2A-B correspond to Fig 6A and 6B, respectively. Connecting lines show paired values of CFU per gram from the spleen or cecum of an individual mouse. (TIF) [file ppat.1008763.s002.tif]

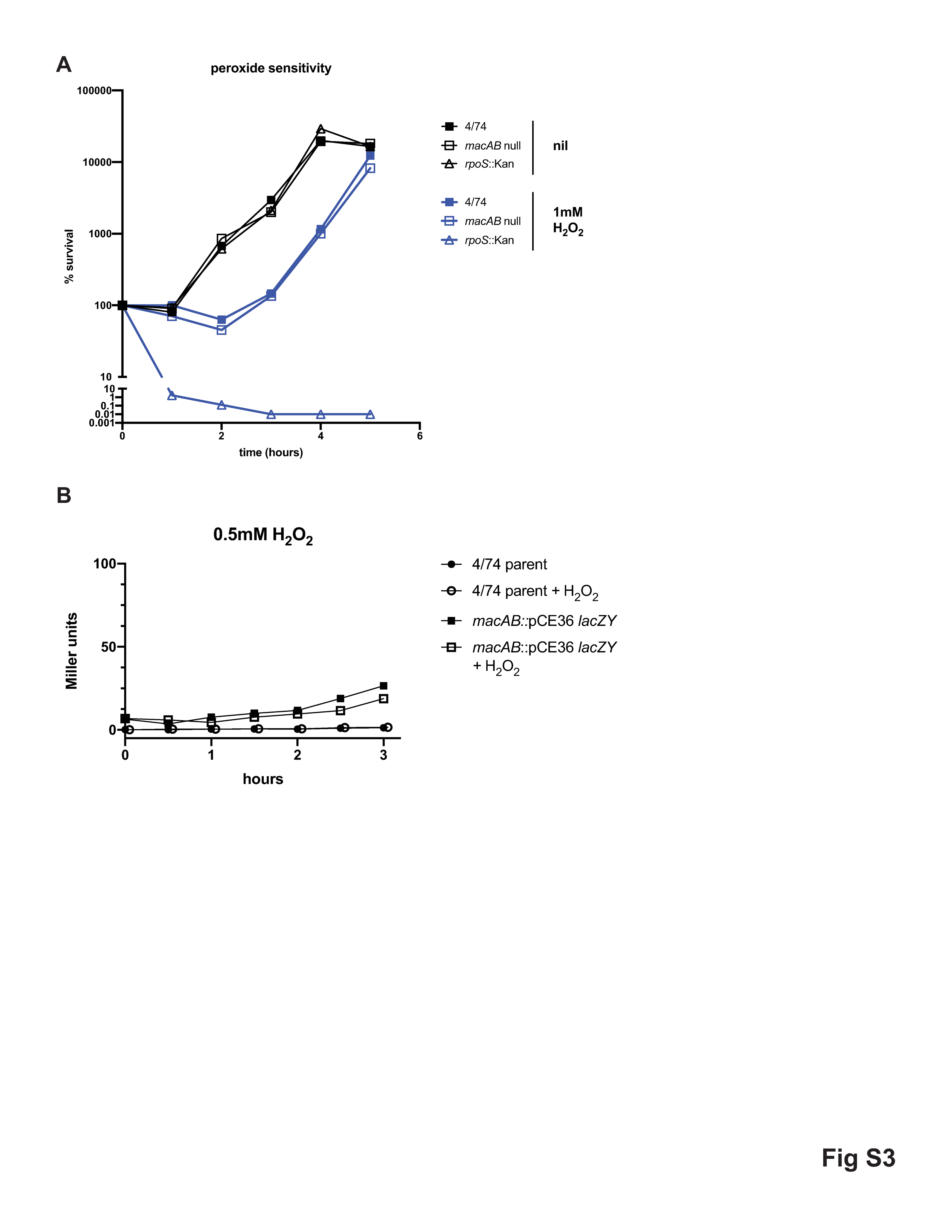

Supplement: S3 Fig — (A) Survival of 4/74 macAB null mutant after peroxide treatment. 4/74, 4/74 macAB null and 4/74 rpoS::Kan were grown in LB Miller overnight with appropriate antibiotics, normalized to OD600 = 1 before 1:100 dilution into fresh LB with or without 1mM H2O2, growing at 37°C while rotating. Cells were removed hourly and serial dilutions plated to calculate percent survival in reference to CFUs at t = 0. (B) Transcriptional induction after peroxide exposure. 4/74 parent and the 4/74 macAB::pCE36 lacZY transcriptional fusion were normalized to OD600 = 1 after overnight culture in LB, followed by 1:100 dilution into fresh LB medium and growth while shaking at 37°C. At OD600 = 0.5 (~2 hours of growth), mid-exponential cells were pelleted and resuspended in the same volume of fresh LB with or without 0.5mM H2O2. Cells were removed every 30 minutes and assayed for β-galactosidase production as described in Materials and Methods. (TIF) [file ppat.1008763.s003.tif]

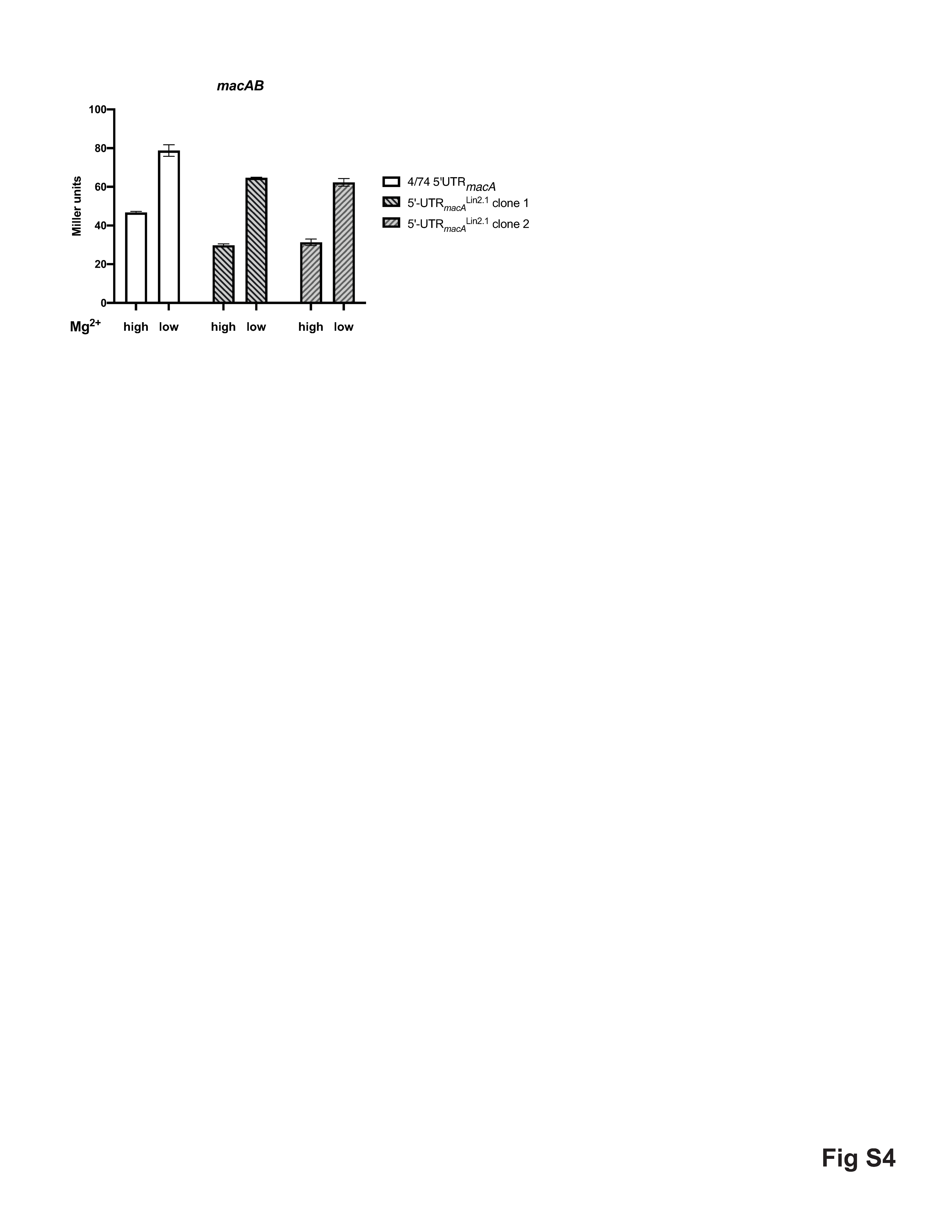

Supplement: S4 Fig — Two clones of 4/74 macAB::pCE36 transcriptional fusion strains with the 5’-UTRmacALin2.1 SNP preceding macA were grown to mid-exponential phase in N minimal medium pH 7.4 with high Mg2+ (10mM) then shifted to the same or low Mg2+ (10μM) media and grown for 90 minutes. β-galactosidase activity was measured using a kinetic Miller assay as described in Materials and Methods. The 5’-UTRmacALin2.1 SNP was incorporated by λ red recombination using the primer pair 1288b, 1289 to amplify the KmR cassette from pKD4. Transcriptional fusions generated with pCE36 include an internal, independent ribosome binding site for translation of lacZY from the transcript. (TIF) [file ppat.1008763.s004.tif]
