# Supplementary material for: Genetic variation in the MacAB-TolC efflux pump influences pathogenesis of invasive Salmonella isolates from Africa
Source: PLoS Pathog. 2020 Aug 24;16(8):e1008763. doi: 10.1371/journal.ppat.1008763 (PMC7446830; doi:10.1371/journal.ppat.1008763)
Supplement: S2 Table — (PDF) [file ppat.1008763.s006.pdf]

Table S2: Bacterial Strains

Bacterial Strain	Description	Reference/ Origin
E.coli strains		
S17-1 λpir	*pro thi hsdR recA* chromosome::RP4-2 Tc::Mu Km::Tn*7*/λ*pir*; Tp^R, Sm^R	(1)
10beta	*endA1* cloning strain	NEB
Salmonella Typhimurium		
4/74	ST19 isolate	(2)
D23580	ST313 Lineage 2 isolate	(3)
4/74 *phoP*::Tn*10*	By P22 phage transduction from SL1344 *phoP*::Tn*10*	This study; (4)
4/74 Δ*macAB*	Markerless by pEMG method with pJH03	This study
4/74 *phoP*::Tn*10* *ΔmacABΔacrAB*	Markerless *macAB acrAB* null by pEMG method with pJH03, then pJH04; *phoP*::Tn*10* by P22 phage	This study
JS198/pCE36	LT2 *metE551 metA22 ilv452 trpB2 hisC527*(am) *galE496 xyl-404 rpsL120 flaA66 hsdL6 hsdSA29* zjg8103::$pir1^+$ recA1 / pCE36; *ahp*	(5)
4/74 *macAB*::pCE36	4/74 *macAB*::pCE36, *lacZY* transcriptional fusion Km^R	This study
4/74 *phoN*::pCE36	4/74 *phoN*::pCE36, *lacZY* transcriptional fusion Km^R	This study
4/74 *phoP::*Tn*10* *macAB*::pCE36	4/74 *phoP*::Tn*10*; macAB::pCE36, *lacZY* transcriptional fusion. Tet^R Km^R	This study
4/74 *phoP*::Tn*10* *phoN*::pCE36	4/74 *phoP*::Tn*10*; *phoN*::pCE36, *lacZY* transcriptional fusion Tet^R Km^R	This study
4/74 *macA*$^{C\to T}$	4/74 modified by pEMG method with plasmid pJH06	This study†
4/74 *macA*$^{C\to T}$*macB*^{indel}	4/74 modified sequentially by pEMG method with plasmids pJH06, pJH07	This study†
4/74 5'-$UTR_{macA}^{Lin2.1}$	4/74 modified by pEMG method with plasmid pJH09	This study
4/74 5'$UTR_{macA}^{Lin2.1}$*macA*$^{C\to T}$	4/74 *macA*$^{C\to T}$ modified by pEMG method with plasmid pJH10	This study
4/74 *orgA*::Tn*5lacZY*	P22 phage transduction from SL1344 *orgA*::Tn*5lacZY*	This study; (6)
4/74 *orgA*::Tn*5lacZY* Δ*ssaV*	P22 phage transduction from SL1344 *orgA*::Tn*5lacZY* followed by pEMG method with plasmid pJH02	This study
4/74 *orgA*::Tn*5lacZY* Δ*ssaV* Δ*macAB*	P22 phage transduction from SL1344 *orgA*::Tn*5lacZY* followed by pEMG method with plasmid pJH02 then pJH03	This study; (6)
D23580 *macA*$^{C\to T}$*macB*^{ST19}	D23580 modified by pEMG method with plasmid pJH07	This study†

D23580 *macA*^{ST19}*macB*^{ST19}	D23580 *macA*$^{C\rightarrow T}$*macB*^{ST19} modified by pEMG method with plasmid pJH05	This study†
D23580 *ΔmacAB*	Markerless by pEMG method with plasmid pJH03	This study
D23580 *ΔorgA*	Markerless by pEMG method with plasmid pJH01	This study
D23580 *ΔorgA ΔssaV*	D23580 *ΔorgA* modified by pEMG method with plasmid pJH02	This study
D23580 *ΔorgA ΔssaV ΔmacAB*	D23580 *ΔorgA ΔssaV* modified by pEMG method with plasmid pJH03	This study
D23580-Kan^{R}	D23580 with Kan^{R} cassette introduced by lambda red recombination using primers del_23_F and del_23_F	This study

† = The nucleotide-engineered strain was genome-sequenced to ensure it contained the correct genetic modification and no other changes (see Methods).

References

1. Simon R, Priefer U, Pühler A. A Broad Host Range Mobilization System for In Vivo Genetic Engineering: Transposon Mutagenesis in Gram Negative Bacteria. Bio/Technology. 1983 Nov;1(9):784–91.

2. Rankin JD, Taylor RJ. The estimation of doses of Salmonella typhimurium suitable for the experimental production of disease in calves. Vet Rec. 1966 May 1;78(21):706–7.

3. Kingsley RA, Msefula CL, Thomson NR, Kariuki S, Holt KE, Gordon MA, et al. Epidemic multiple drug resistant Salmonella Typhimurium causing invasive disease in sub-Saharan Africa have a distinct genotype. Genome Res. 2009 Dec 1;19(12):2279–87.

4. Rathman M, Sjaastad MD, Falkow S. Acidification of phagosomes containing Salmonella typhimurium in murine macrophages. Infect Immun. 1996 Jul 1;64(7):2765–73.

5. Ellermeier CD, Janakiraman A, Slauch JM. Construction of targeted single copy lac fusions using λ Red and FLP-mediated site-specific recombination in bacteria. Gene. 2002 May 15;290(1):153–61.

6. Penheiter KL, Mathur N, Giles D, Fahlen T, Jones BD. Non-invasive Salmonella typhimurium mutants are avirulent because of an inability to enter and destroy M cells of ileal Peyer's patches. Mol Microbiol. 1997;24(4):697–709.
