# Supplementary material for: Genetic variation in the MacAB-TolC efflux pump influences pathogenesis of invasive Salmonella isolates from Africa
Source: PLoS Pathog. 2020 Aug 24;16(8):e1008763. doi: 10.1371/journal.ppat.1008763 (PMC7446830; doi:10.1371/journal.ppat.1008763)
Supplement: S3 Table — (PDF) [file ppat.1008763.s007.pdf]

Table S3: Plasmids

Plasmid ID	Plasmid Name	Description	Reference/Origin
pKD4	pKD4	*aph*-cassette template plasmid; Km^R	(1)
pSIM5-Tet	pSIM5-Tet	λ Red recombination plasmid, temperature-inducible; Tc^R	(2)
pSW-2	pSW-2	I-SceI nuclease inducible by *m*-toluate; Gm^R	(3)
pEMG	pEMG	R6K-origin suicide plasmid; Km^R	(3)
pJH01	pEMG::*orgA*del	800bp flanking regions of *orgA* for deletion	This study
pJH02	pEMG::*ssaV*del	800bp flanking regions of *ssaV* for deletion	This study
pJH03	pEMG::*macAB*del	800bp flanking regions of *macAB* for deletion	This study
pJH04	pEMG::*acrAB*del	800bp flanking regions of *acrAB* for deletion	This study
pJH05	pEMG::$macA^{ST19}$	From 4/74 gDNA template	This study
pJH06	pEMG::$macA^{C\rightarrow T}$	From D23580 gDNA template	This study
pJH07	pEMG::$macB^{ST19}$	From 4/74 gDNA template	This study
pJH08	pEMG::$macB^{indel}$	From D23580 gDNA template	This study
pJH09	pEMG::5'-$UTR_{macA}^{Lin2.1}$	Mutagenesis of pJH05 template by PCR and HiFi DNA assembly	This study
pJH10	pEMG:: 5'-$UTR_{macA}^{LinII.1}macA^{C\rightarrow T}$	Mutagenesis of pJH06 template by PCR and HiFi DNA assembly	This study
pBAD33.1	pBAD33.1	Arabinose inducible expression plasmid; p15A origin, Cm^R	Addgene #36267; (4)
pJH11	pBAD33.1 - $macA^{ST19}macB^{ST19}$	PCR product of 1223, 1224 from 4/74 ligated into pBAD33.1	This study
pJH12	pBAD33.1 - $macA^{C\rightarrow T}macB^{ST19}$	PCR product of 1223, 1224 from 4/74 $macA^{C\rightarrow T}$ ligated into pBAD33.1	This study
pJH13	pBAD33.1 - $macA^{C\rightarrow T}macB^{indel}$	PCR product of 1223, 1224 ligated into pBAD33.1	This study
pACYC177	pACYC177	p15A origin, Amp^R Km^R	(5)
pJH15	pACYC177-$macA^{ST19}macB^{ST19}$	T7 RBS - $macA^{ST19}macB^{ST19}$ 2xTerm of pJH11 inserted after the *Amp* promoter into ScaI-PstI digested pACYC177; Km^R	This study
pJH16	pACYC177-$macA^{C\rightarrow T}macB^{ST19}$	T7 RBS - $macA^{ST19}macB^{ST19}$ 2xTerm of pJH12 inserted after the *Amp* promoter into ScaI-PstI digested pACYC177; Km^R	This study

pJH17	pACYC177-*macA*$^{C\rightarrow T}$*macB*^{indel}	T7 RBS - *macA*^{ST19}*macB*^{ST19} 2xTerm of pJH13 inserted after the *Amp* promoter into ScaI-PstI digested pACYC177; Km^R	This study

References

1. Datsenko KA, Wanner BL. One-step inactivation of chromosomal genes in Escherichia coli K-12 using PCR products. Proc Natl Acad Sci. 2000 Jun 6;97(12):6640–5.

2. Koskiniemi S, Pränting M, Gullberg E, Näsvall J, Andersson DI. Activation of cryptic aminoglycoside resistance in Salmonella enterica. Mol Microbiol. 2011 Jun;80(6):1464–78.

3. Martínez-García E, de Lorenzo V. Engineering multiple genomic deletions in Gram-negative bacteria: analysis of the multi-resistant antibiotic profile of Pseudomonas putida KT2440. Environ Microbiol. 2011 Oct 1;13(10):2702–16.

4. Chung HS, Raetz CRH. Interchangeable Domains in the Kdo Transferases of Escherichia coli and Haemophilus influenzae. Biochemistry. 2010 May 18;49(19):4126–37.

5. Chang AC, Cohen SN. Construction and characterization of amplifiable multicopy DNA cloning vehicles derived from the P15A cryptic miniplasmid. J Bacteriol. 1978 Jun 1;134(3):1141–56.
