# Supplementary material for: Genetic variation in the MacAB-TolC efflux pump influences pathogenesis of invasive Salmonella isolates from Africa
Source: PLoS Pathog. 2020 Aug 24;16(8):e1008763. doi: 10.1371/journal.ppat.1008763 (PMC7446830; doi:10.1371/journal.ppat.1008763)
Supplement: S4 Table — (PDF) [file ppat.1008763.s008.pdf]

Table S4: Primers

Name	Sequence (Restriction sites underlined)	Reference
1223 *macAB* NdeI FWD	TACTACATATGATGCGTGCTAAGGGAAAGAAATT	This study
1224 *macAB* HindIII REV	TACTAAAGCTTTTACTCCCTTGCCAGCGCAT	This study
1272 *macAB* ScaI FWD	TACTAAGTACTAACCCGGGGATCCTCTAGAAAT	This study
1273 *macAB* PstI REV	TACTACTGCAGGTTTGTAGAAACGCAAAAAGG	This study
1288 *macAB* FWD	CGGTTTACTTTCTGGTTAAAATTTTGCCGTCAGGGTTTCTGTGTAGGCTGGAGCTGCTTC	This study
1288b *macAB* 5'-UTR$_{macA}^{Lin2.1}$	CGGTTTACTTTCTGGTTAAGATTTTGCCGTCAGGGTTTCTGTGTAGGCTGGAGCTGCTTC	This study
1289 *macAB* REV	CTGTTGCATCTTATCAGGCCGACAAATGACGTCAGCAAGACATATGAATATCCTCCTTAG	This study
1303 PS1	TGTAGGCTGGAGCTGCTTCG	(1)
1305 Lac	CATATGAATATCCTCCTTAG	(1)
1309 prSNPmacA TS1 FWD	TCTGAATTCGAGCTCGGTACGACTTGCGCAAACTGACAGA	This study
1310 prSNPmacA TS1 REV	GAAACCCTGACGGCAAAATCTTAACCAGAAAGTAAACCGTATCT	This study
1311 prSNPmacA TS2 FWD	CGGTTTACTTTCTGGTTAAGATTTTGCCGTCAGGGTTTCT	This study
1312 prSNPmacA TS2 REV	TGCCTGCAGGTCGACTCTAGAGAATATCTTTTAAAACGCCTTCA	This study
del_D23_F	CATTCAAGATGTGAATGTAATTTATTTGTTTATAATGTTATTAATATATGGTGTAGGCTGGAGCTGCTTC	This study
del_D23_R	GAAAATATAAAAGACAAAACAATCATTAAAACATTTATCAACTTACATTACATATGAATATCCTCCTTA	This study
Rv_STM4196-7_ext	GGGAATACTGGCGACATG	This study
Fw_STM4196-7_ext	TAGTTTTGCCCACCATCCT	This study

Reference

1. Ellermeier CD, Janakiraman A, Slauch JM. Construction of targeted single copy lac fusions using λ Red and FLP-mediated site-specific recombination in bacteria. Gene. 2002 May 15;290(1):153–61.
